# Dietary intake of advanced glycation endproducts and risk of hepatobiliary cancers: A multinational cohort study

**DOI:** 10.1002/ijc.33612

**Published:** 2021-05-06

**Authors:** Ana‐Lucia Mayén, Elom K. Aglago, Viktoria Knaze, Reynalda Cordova, Casper G. Schalkwijk, Karl‐Heinz Wagner, Krasimira Aleksandrova, Veronika Fedirko, Pekka Keski‐Rahkonen, Michael F. Leitzmann, Verena Katzke, Bernard Srour, Matthias B. Schulze, Giovanna Masala, Vittorio Krogh, Salvatore Panico, Rosario Tumino, Bas Bueno‐de‐Mesquita, Magritt Brustad, Antonio Agudo, María Dolores Chirlaque López, Pilar Amiano, Bodil Ohlsson, Stina Ramne, Dagfinn Aune, Elisabete Weiderpass, Mazda Jenab, Heinz Freisling

**Affiliations:** ^1^ Nutrition and Metabolism Branch International Agency for Research on Cancer (IARC‐WHO) Lyon France; ^2^ Department of Nutritional Sciences University of Vienna Vienna Austria; ^3^ Department of Internal Medicine, CARIM School for Cardiovascular Diseases Maastricht University Medical Center Maastricht The Netherlands; ^4^ Immunity and Metabolism Department of Nutrition and Gerontology German Institute of Human Nutrition Potsdam‐Rehbruecke Nuthetal Germany; ^5^ Rollins School of Public Health Emory University Atlanta Georgia USA; ^6^ Department of Epidemiology and Preventive Medicine University of Regensburg Regensburg Germany; ^7^ Division of Cancer Epidemiology Germany Cancer Research Center (DKFZ) Heidelberg Germany; ^8^ Department of Molecular Epidemiology German Institute of Human Nutrition Potsdam‐Rehbruecke Nuthetal Germany; ^9^ Institute of Nutrition Science University of Potsdam Nuthetal Germany; ^10^ Cancer Risk Factors and Life‐Style Epidemiology Unit Institute for Cancer Research Prevention and Clinical Network—ISPRO Florence Italy; ^11^ Epidemiology and Prevention Unit Fondazione IRCCS Istituto Nazionale dei Tumori di Milano Milan Italy; ^12^ Dipartimento di Medicina Clinica e Chirurgia Federico II University Naples Italy; ^13^ Cancer Registry and Histopathology Department Provincial Health Authority (ASP) Ragusa Italy; ^14^ Department for Determinants of Chronic Diseases (DCD) National Institute for Public Health and the Environment (RIVM) Bilthoven The Netherlands; ^15^ Department of Community Medicine UiT The Arctic University of Norway Tromsø Norway; ^16^ Unit of Nutrition and Cancer Catalan Institute of Oncology—ICO, Nutrition and Cancer Group, Bellvitge Biomedical Research Institute—IDIBELL Barcelona Spain; ^17^ Department of Epidemiology Regional Health Council, IMIB‐Arrixaca Murcia Spain; ^18^ CIBER Epidemiología y Salud Pública (CIBERESP) Madrid Spain; ^19^ Department of Health and Social Sciences University of Murcia Murcia Spain; ^20^ Ministry of Health of the Basque Government, Public Health Division of Gipuzkoa BioDonostia Health Research Institute Donostia‐San Sebastian Spain; ^21^ Department of Internal Medicine Lund University, Skåne University Hospital Malmö Sweden; ^22^ Nutritional Epidemiology, Department of Clinical Sciences Malmö Lund University Malmö Sweden; ^23^ Department of Epidemiology and Biostatistics, School of Public Health Imperial College London London UK; ^24^ Department of Nutrition Bjørknes University College Oslo Norway; ^25^ Department of Endocrinology, Morbid Obesity and Preventive Medicine Oslo University Hospital Oslo Norway

**Keywords:** advanced glycation endproducts, bile duct cancers, EPIC study, gallbladder cancer, hepatocellular carcinoma

## Abstract

Advanced glycation endproducts (AGEs) may contribute to liver carcinogenesis because of their proinflammatory and prooxidative properties. Diet is a major source of AGEs, but there is sparse human evidence on the role of AGEs intake in liver cancer etiology. We examined the association between dietary AGEs and the risk of hepatobiliary cancers in the European Prospective Investigation into Cancer and Nutrition prospective cohort (n = 450 111). Dietary intake of three AGEs, N^ε^‐[carboxymethyl]lysine (CML), N^ε^‐[1‐carboxyethyl]lysine (CEL) and N^δ^‐[5‐hydro‐5‐methyl‐4‐imidazolon‐2‐yl]‐ornithine (MG‐H1), was estimated using country‐specific dietary questionnaires linked to an AGEs database. Cause‐specific hazard ratios (HR) and their 95% confidence intervals (CI) for associations between dietary AGEs and risk of hepatocellular carcinoma (HCC), gallbladder and biliary tract cancers were estimated using multivariable Cox proportional hazard regression. After a median follow‐up time of 14.9 years, 255 cases of HCC, 100 cases of gallbladder cancer and 173 biliary tract cancers were ascertained. Higher intakes of dietary AGEs were inversely associated with the risk of HCC (per 1 SD increment, HR‐_CML_ = 0.87, 95% CI: 0.76‐0.99, HR‐_CEL_ = 0.84, 95% CI: 0.74‐0.96 and HR‐_MH‐G1_ = 0.84, 95% CI: 0.74‐0.97). In contrast, positive associations were observed with risk of gallbladder cancer (per 1 SD, HR‐_CML_ = 1.28, 95% CI: 1.05‐1.56, HR‐_CEL_ = 1.17; 95% CI: 0.96‐1.40, HR‐_MH‐G1_ = 1.27, 95% CI: 1.06‐1.54). No associations were observed for cancers of the intra and extrahepatic bile ducts. Our findings suggest that higher intakes of dietary AGEs are inversely associated with the risk of HCC and positively associated with the risk of gallbladder cancer.

## Abstract 2

What's new?

Advanced glycation endproducts (AGEs) are proteins or lipids with sugars added to them, and they can form in foods during cooking. They have pro‐inflammatory and pro‐oxidative properties in the body. These authors investigated whether dietary consumption of AGEs affect risk of liver cancer, using the European Prospective Investigation into Cancer and Nutrition (EPIC) cohort. Participants filled out food questionnaires to determine AGE consumption. Higher intakes of AGEs were associated with reduced risk of hepatocellular carcinoma (HCC) and increased risk of gallbladder cancer. However, it's still not entirely clear how much AGEs in the diet contribute to circulating AGE levels.

**Abbreviations**
AGEs: advanced glycation endproducts
BMI: body mass index
CEL: N^ε^‐[1‐carboxyethyl]lysine
CI: confidence intervals
CML: N^ε^‐[carboxymethyl]lysine
DQ: dietary questionnaires
EPIC: European Prospective Investigation into Cancer and Nutrition
HCC: hepatocellular carcinoma
HR: hazard ratios
MG‐H1: N^δ^‐[5‐hydro‐5‐methyl‐4‐imidazolon‐2‐yl]‐ornithine
RAGE: receptor of AGEs

## 1 INTRODUCTION

Liver cancer was the sixth most common cancer and the fourth leading cause of cancer‐related death worldwide in 2018.^1^ The most common type of liver cancer is hepatocellular carcinoma (HCC).^2^ High‐risk regions for HCC are usually located in low‐ and middle‐income countries, where the main prevailing causes are hepatitis infections and high exposure to aflatoxins.^2^ However, HCC rates are on the rise in higher‐income countries, where hepatitis and aflatoxin exposure are less common.^3^, ^4^ Purported causes for the increasing HCC rates in traditionally low‐risk countries include the rising prevalence of obesity, Type 2 diabetes and nonalcoholic fatty liver disease.^5^, ^6^, ^7^ Obesity and associated metabolic disorders are in part related to long‐term unhealthy lifestyle and dietary choices, such as overconsumption of energy‐dense and thermally processed foods.^8^

Foods that undergo prolonged high‐heat processing, such as deep frying, grilling or broiling, are rich sources of advanced glycation endproducts (AGEs).^9^ AGEs are formed by the reaction between reducing sugars and proteins, and enhance flavor, smell and appearance of food, but represent also a class of prooxidants in foods.^10^ The best characterized dietary AGEs include N^ε^‐[carboxymethyl]lysine (CML), N^ε^‐[1‐carboxyethyl]lysine (CEL) and N^δ^‐[5‐hydro‐5‐methyl‐4‐imidazolon‐2‐yl]‐ornithine (MG‐H1).^11^ Approximately 10% of ingested AGEs are thought to be absorbed in the gastrointestinal tract and around 30% of the absorbed amount is excreted through the kidneys^12^ while the rest remains in the body.^13^ The liver is the main exposure organ of absorbed dietary AGEs and specialized liver cells, in particular endothelial and Kupffer cells, play an important role in clearing AGEs from the circulation.^14^ AGEs are also produced endogenously during normal metabolism and irreversibly accumulate with age in several types of body tissues, in particular in a state of impaired glucose tolerance, and it remains unclear how much the dietary AGEs contribute to the total amount of AGEs in the body.^12^

In rodent studies, higher dietary AGEs administration led to liver tissue deposition and increased hepatic expression of receptor of AGEs (RAGE).^15^ The binding of AGEs to RAGE triggers oxidative stress and chronic/acute inflammation.^16^ It has also been reported that ingested dietary AGEs can accumulate in the gallbladder.^10^ Furthermore, several human and experimental studies have shown an association of higher AGEs exposure with hepatic disorders—from minor steatosis to hepatic cirrhosis.^15^ The literature also suggests a link between AGE‐RAGE binding and malignant transformations of hepatic cells.^17^ Dietary AGEs may therefore play an important role in the development of cancers of the liver. In comparison to other chronic diseases such as diabetes or cardiovascular disease,^18^ the potential relationship between dietary AGEs and cancer risk remains an emerging field of research.^19^

In our study, using the large multinational European Prospective Investigation into Cancer and Nutrition (EPIC) cohort, we investigated the association between dietary intake of AGEs and the risk of HCC and other hepatobiliary cancers.

## 2 MATERIALS AND METHODS

### 2.1 Study population

EPIC is a prospective cohort study of 521 324 participants that aims to assess cancer and other chronic disease risk factors. Participants in EPIC were recruited in 23 centers located in 10 European countries (Denmark, France, Germany, Greece, Italy, the Netherlands, Norway, Spain, Sweden and the United Kingdom).^20^, ^21^ The study design and methodology for recruitment have been previously described.^20^, ^21^, ^22^ Briefly, participants were recruited from 1992 to 2000. Participants were representative of the general population except for France (female school employees participating in the national health insurance scheme), Utrecht and Florence (women from the breast cancer screening programs), Oxford (vegetarian/vegan volunteers) and some centers from Italy and Spain (blood donors).

For the current study, among the 521 324 participants recruited at baseline, 25 184 (5%) were excluded because they were prevalent cancer cases, 4148 (1%) were missing information on follow‐up and 6259 (1%) because of missing information on lifestyle or dietary information. Also, 9573 (2%) participants in the top and bottom 1% of the total energy intake to estimated energy requirements ratio were excluded. After excluding Greece (no data provided for our study) and one participant from Bilthoven, who withdrew participation in EPIC, a total of 450 111 participants were eligible for the study (Figure S1).

### 2.2 Identification and follow‐up of cases

In most countries, cases were identified through population cancer registries (Denmark, Italy, the Netherlands, Norway, Spain, Sweden and the United Kingdom). In other countries such as France, Germany and Naples (Italy), a combination of methods was used to identify cases including health insurance records, cancer and pathology registries and an active follow‐up of study participants. Cancer incidence data were coded according to the 10th revision of the International Statistical Classification of Diseases, Injuries and Causes of Death and the second revision of the International Classification of Diseases for Oncology. Only first incident cases were included according to topographical codes: HCC (C22.0), intrahepatic bile duct (C22.1), extrahepatic bile duct (C24.0) and gallbladder (C23.9). For each identified case, the histology and the methods used for diagnosis were reviewed and metastatic cases or other types of primary liver cancer were excluded.

### 2.3 Dietary assessment and estimation of AGE intake

In EPIC, country‐ or center‐specific validated dietary questionnaires (DQ) were used at baseline, accounting for the usual food intake during the previous 12 months.^20^ In Umeå (Sweden), Denmark, Norway and Naples (Italy), semiquantitative food frequency questionnaires were used. Malmö (Sweden) and the United Kingdom used semiquantitative food frequency questionnaires in combination with 7‐day and 14‐day records, respectively. Netherlands, Germany, Northern Italy and France used quantitative DQ. In Spain and Ragusa (Italy) the quantitative DQ were interviewer‐administered and structured by meals. Harmonization of food groups and portion sizes for quantification was carried out centrally at the International Agency for Research on Cancer.^23^

A reference dietary AGEs composition database was used, which contain CML, CEL and MG‐H1 concentrations (in mg/100 g of food) obtained using ultra‐performance liquid chromatography‐tandem mass spectrometry analysis of 190 food items commonly consumed in Europe.^11^ Foods from the reference database were matched to DQ foods by name and descriptors, particularly those pertaining to preparation and processing whenever applicable. When matching, any generic or multi‐ingredient DQ foods were decomposed into more specific foods or ingredients based on country‐specific recipes obtained from previous EPIC projects.^23^, ^24^ The EPIC AGEs composition database was then generated and, in turn, used to obtain the daily intake (mg/d) of CML, CEL and MG‐H1 per study participant. Estimated associations between higher dietary intakes of any of three AGEs and greater weight gain after an average of 5 years of follow‐up in the same study population were as expected,^24^ which confirms face validity of these data.

Lifestyle questionnaires were used to obtain information on education, physical activity, lifetime alcohol intake, smoking status, duration and intensity and self‐reported diabetes mellitus status. Anthropometric measures were assessed at recruitment and body mass index (BMI) was computed as weight in kg over height in square meters.

### 2.4 Statistical analysis

Dietary intakes of CML, CEL and MG‐H1 were natural log‐transformed and total energy intake adjusted. For energy adjustment, we computed standardized residuals of each of the three AGEs by regressing the ln‐transformed AGEs on total energy intake and center. These energy‐adjusted residuals of AGEs were analyzed separately on a continuous scale per SD increment and as tertiles of intake combining men and women across all centers.

We used Cox proportional hazards models to estimate cause‐specific hazard ratios (HR) and 95% confidence intervals (CI). Entry time was age at recruitment and exit time was either age at diagnosis, death or censoring date (lost to or end of follow‐up), whichever event came first. The proportional hazards assumption for all variables in the model was tested with Schoenfeld residuals.^25^ Reported *P*‐values are two‐sided and values lower than .05 were considered statistically significant.

Two main models were fitted with different sets of adjustments. Model 1 was stratified by center, sex and age at recruitment (in 1‐year categories), and adjusted for total energy intake (continuous, kcal/d). Model 2 was further adjusted for BMI (continuous, kg/m^2^), smoking intensity (never, currently smokes 1‐15 cigarettes/d, currently smokes 16‐25 cigarettes/d, currently smokes 26+ cigarettes/d, former smoker who quit less than 10 years ago, former smoker who quit 10‐20 years ago, former smoker who quit more than 20 years ago, current occasional smoker of pipes or cigars, and missing), baseline alcohol intake (continuous using restricted cubic splines to account for nonlinearity, g/d), pattern of lifetime alcohol intake (light drinkers [never drinkers, former light and heavy drinkers, light drinkers and never heavy drinkers], heavy drinkers [periodically and always heavy drinkers] and missing), the Cambridge physical activity index^26^ (inactive, moderately inactive, moderately active, active and missing), highest attained educational level (none, primary completed, technical/professional, secondary, tertiary and missing), coffee intake (continuous, g/d), self‐reported prevalent diabetes mellitus (no, yes, missing) and dietary fiber intake (continuous, g/d). We also computed the sum of the three AGEs by summing the standardized residuals of each AGE. Participants with missing values were included in analyses and respective variables were coded with a missing value indicator, unless otherwise specified. Proportions of missing values for each variable are reported in Table 1.

**TABLE 1:** Baseline characteristics of the study population according to tertiles of dietary intake of advanced glycation endproducts (AGEs)^a^ in the European Prospective Investigation into Cancer and Nutrition, 1992‐2000 (n = 450 111)

Characteristics	CML			CEL			MG‐H1		
	T1	T2	T3	T1	T2	T3	T1	T2	T3
AGE consumption, mg/d^b^	2.3 ± 1.0^b^	3.0 ± 1.0	4.0 ± 1.3	1.7 ± 0.7	2.1 ± 0.7	2.8 ± 1.0	15.5 ± 6.0	20.5 ± 6.3	29.1 ± 10.7
Age at recruitment, years	51.4 ± 9.7	50.6 ± 9.7	51.4 ± 9.9	50.9 ± 10.1	51.1 ± 9.5	51.4 ± 9.6	51.3 ± 9.8	50.6 ± 9.7	51.5 ± 9.7
BMI, kg/m^b^	25.2 ± 4.2	25.3 ± 4.2	25.3 ± 4.2	25.0 ± 4.1	25.3 ± 4.1	25.5 ± 4.3	25.3 ± 4.2	25.3 ± 4.2	25.2 ± 4.2
Women, %	68	71	73	70	71	71	68	72	72
Dietary variables									
Total energy, kcal/d	2070 ± 734	2081 ± 586	2078 ± 517	2077 ± 735	2087 ± 583	2066 ± 518	2065 ± 723	2091 ± 588	2074 ± 529
Coffee, g/d^c^	217 ± 215	207 ± 197	190 ± 190	194 ± 198	213 ± 203	207 ± 202	195 ± 193	209 ± 205	210 ± 205
Fiber, g/d^c^	11 ± 3	11 ± 3	11 ± 3	11 ± 4	11 ± 3	11 ± 3	10 ± 3	11 ± 3	12 ± 3
Mediterranean diet score^d^	8.8 ± 3.1	8.6 ± 3.0	8.4 ± 2.8	8.7 ± 3.1	8.5 ± 2.9	8.6 ± 2.9	8.1 ± 3.1	8.8 ± 3.0	8.9 ± 2.9
Alcohol at recruitment, g/d	17 ± 22	11 ± 14	8 ± 11	15 ± 21	11 ± 15	9 ± 12	16 ± 22	11 ± 14	9 ± 12
Lifetime alcohol intake, %									
Light drinkers	60	66	71	62	65	69	61	67	69
Heavy drinkers	16	7.7	5.0	14	8.1	6.8	14	8.0	6.2
Missing	25	27	24	24	27	24	25	25	25
Self‐reported diabetes, %									
Yes	2.3	2.2	2.7	2.0	2.1	3.0	2.1	2.2	2.9
Physical activity, %									
Inactive	20	19	20	20	18	20	21	19	19
Moderately inactive	33	33	34	33	33	34	34	33	33
Moderately active	26	27	27	26	28	27	26	27	27
Active	19	19	18	19	19	18	18	18	19
Missing	2.0	2.1	1.8	1.8	2.2	1.9	1.8	2.1	2.0
Smoking intensity, %									
Never	38	43	47	41	42	44	40	43	45
Current, 1–15 cigarettes/d	13	12	10	12	12	11	13	12	10
Current, 16–25 cigarettes/d	7.5	6.0	4.9	6.7	6.3	5.5	7.6	6.2	4.6
Current, 26+ cigarettes/d	2.1	1.2	1.1	1.8	1.3	1.3	2.1	1.4	1.0
Former, quit less 10 years ago	10.3	9.7	8.9	9.6	9.9	9.4	9.7	9.8	9.4
Former, quit 11–20 years ago	8.6	8.5	8.0	8.3	8.4	8.4	8.1	8.5	8.5
Former, quit 20+ years ago	8.0	8.1	8.4	7.7	8.5	8.4	7.8	8.0	8.8
Current, pipe, cigar use	9.1	8.4	9.1	9.3	7.9	9.4	9.1	8.4	9.1
Missing	3.5	3.1	3.0	3.3	3.2	3.0	3.4	3.0	3.2
Education, %									
None	3.5	3.1	3.8	4.0	2.8	3.6	3.3	3.5	3.6
Primary completed	25	24	25	24	25	25	25	25	24
Technical/professional	23	24	23	22	25	23	23	24	23
Secondary	21	21	21	21	21	21	20	21	21
Tertiary	25	24	24	26	24	23	24	24	25
Missing	4.0	3.3	4.0	3.5	3.5	4.3	4.4	3.3	3.6
Hepatitis B and C, %^e^									
Yes	18	27	17	21	22	18	22	16	21

Abbreviations: BMI, body mass index; CEL, N^ε^‐[1‐carboxyethyl]lysine; CML, N^ε^‐[carboxymethyl]lysine; MG‐H1, N^δ^‐[5‐hydro‐5‐methyl‐4‐imidazolon‐2‐yl]‐ornithine.

^a^ Residuals were computed by a linear regression of the log transformed intake of AGEs, energy and center.

^b^ Mean ± SD, all such values.

^c^ Refers to grams of daily intake per 1000 kcal.

^d^ Ranges from 0 to 18 points, zero showing no adherence to the Mediterranean Diet Pattern.

^e^ Percentages using the nested case‐control dataset with n = 204 cases and n = 205 matched HCC controls.

To evaluate the shape and linearity of associations for continuous intakes of AGEs, three‐knot restricted cubic spline models were fitted at Harrell's default percentiles^27^ (ie, 10th, 50th and 90th) in combination with a Wald‐type test for nonlinearity.^27^

To test whether associations with dietary AGEs were modified by a priori defined covariates, we repeatedly ran our fully adjusted model with a cross‐product term between AGEs and potential effect modifiers (multiplicative interaction corrected for multiple tests using Bonferroni): sex, BMI (normal, overweight, obese), smoking status (never, former, current, missing), diabetes (yes, no, missing), lifetime alcohol consumption pattern (light drinkers, heavy drinkers, missing), study region (North: Sweden, Denmark and Norway; Central: France, the United Kingdom, the Netherlands and Germany; South: Italy and Spain), median age at recruitment (below or above 51.5 years) and dietary energy intake misreporting according to Goldberg cut‐offs (under‐reporters, plausible reporters, overreporters).^28^ We used likelihood ratio tests to assess statistical significance for each interaction. We further fitted model 2 separately in each country for HCC and gallbladder cancer and combined risk estimates by random effects meta‐analysis, and assessed heterogeneity of associations across countries using the *I* ^2^ score.^29^ Due to a small number of cases in France, Norway and the Netherlands, we combined France with Spain, Norway with Sweden and the Netherlands with Germany. We chose the combined neighboring countries, which we assumed to have more similar eating habits.

### 2.5 Sensitivity analysis

To ensure the robustness of our findings, we conducted the following sensitivity analyses for hepatocellular and gallbladder cancers. First, to minimize the influence of reverse causation, we excluded cancer events that occurred during the first 2 years of follow‐up. Second, to account for healthy dietary habits, which may confound associations between dietary AGE intake and cancer risk, we adjusted for the modified relative Mediterranean Diet Score, instead of dietary fiber.^30^ A higher score in the Mediterranean Diet Score indicates higher intakes of vegetables, legumes, fruit and nuts/seeds, cereals, fish and seafood, olive oil; a moderate alcohol consumption; and lower intakes of meat/meat products and dairy products. A number of these food groups are also sources of dietary AGEs. Third, to evaluate potential residual confounding by smoking and heavy alcohol consumption, we excluded in turn current smokers and participants reporting heavy alcohol consumption at any point in time. Fourth, we evaluated whether dietary misreporting may have biased our estimates by adjusting for plausibility of dietary intake reporting based on Goldberg's cut‐off points.^28^ Fifth, we compared results obtained using a missing value indicator with those using a complete case analysis after excluding all subjects with missing values in any of the covariates (total n = 11.1%). Sixth (for hepatocellular cancer only), we used data from the EPIC nested case‐control study to adjust associations for hepatitis virus B and C (HBV/HCV) infection status (measured by HBsAg and anti‐HCV assays) and liver function biomarkers (gamma‐glutamyltransferase, GGT; alanine aminotransferase, ALT; aspartate aminotransferase, AST; alkaline phosphatase, ALP; total bilirubin and albumin) available for a subset of participants.^31^ Odds ratios (OR) for HCC were estimated from multivariable conditional logistic regression, adjusted for matching variables (age, sex, recruitment center, fasting status, time of blood draw and hormonal factors in women), further adjusted for all variables of model 2 (see above), and with additional adjustment for in turn HBC/HCV status and liver function (Table S6). Last, we adjusted for main food sources of dietary AGEs (cereals, red and processed meats and cakes and biscuits). All analyses were performed using STATA 14.2 (Stata Corporation, College Station, TX).

## 3 RESULTS

### 3.1 Characteristics of the study population

The baseline characteristics of the study population by tertiles of dietary intake of AGEs are shown in Table 1. Individuals with the highest intake of AGEs consumed on average less alcohol at recruitment, were less frequently lifetime heavy drinkers, and were more likely to be female (except for CEL intake), have never smoked, and have self‐reported diabetes. During a median follow‐up time of 14.9 years, 255 primary first‐incident HCC cases, 100 gallbladder cases, 88 intrahepatic bile duct cases and 85 extrahepatic bile duct cases were ascertained.

### 3.2 Dietary sources of AGEs

The food sources of CML, CEL and MG‐H1 of the study population are shown in Figure S2. The top four food groups contributing to CML intake were cereals and cereal products (35%), meat and meat products (19%), cakes and biscuits (14%) and dairy (11%). Similarly, the top food groups contributing to CEL intake were meat and meat products (30%), cereals and cereal products (24%), cakes and biscuits (10%) and fish (7%), and for MG‐H1 cereals and cereal products (45%), cakes and biscuits (12%), meat and meat products (12%) and nonalcoholic beverages (4%).

The consumption of food groups by tertiles of dietary intake of AGEs is shown in Table S1. In general, participants in the highest tertile vs lowest tertile had markedly higher consumption of food groups that were major sources of AGEs. This was not the case for dairy, where participants in the highest tertile of CEL or MG‐H1 intake had the lowest intake of dairy.

The percentage contribution of food groups to CML, CEL and MG‐H1 by geographical regions are presented in Figure S3. The highest consumption of CML coming from cereals and cereal products, and meat and meat products was found in the northern region, followed by the southern region, and the central region. The highest intake of CEL and MG‐H1 coming from cereals and cereal products was found in the northern region while the highest intake of CEL and MG‐H1 coming from meat and meat products was found in the southern region.

### 3.3 Estimated dietary intake of AGEs and risk of hepatocellular cancer

In minimally adjusted model 1 (energy adjusted and stratified by sex, center and age), each of the three AGEs was inversely associated with the risk of HCC, with HR corresponding to 0.76 (95% CI, 0.67‐0.85), 0.76 (95% CI, 0.67‐0.86), and 0.70 (95% CI, 0.62‐0.79) per 1 SD increment in dietary intake of CML, CEL and MG‐H1, respectively (Table 2).

**TABLE 2:** Hazard ratios (95% confidence intervals) for hepatobiliary cancer subsites associated with energy‐adjusted intake of advanced glycation endproducts (AGEs)^a^ in the European Prospective Investigation into Cancer and Nutrition, 1992‐2000 (n = 450 111)

	HCC^b^	*P*‐value	Intrahepatic bile duct^b^	*P*‐value	Extrahepatic bile duct^b^	*P*‐value	Gallbladder^b^	*P*‐value
CML								
Cases, n	n = 255		n = 88		n = 85		n = 100	
Model 1^c^	0.76 (0.67‐0.85)	<.001	0.86 (0.70‐1.05)	.139	1.06 (0.85‐1.32)	.617	1.30 (1.07‐1.57)	.008
Model 2^d^	0.87 (0.76‐0.99)	.030	0.88 (0.71‐1.09)	.230	1.08 (0.86‐1.37)	.506	1.28 (1.05‐1.56)	.014
CEL								
Cases, n	n = 255		n = 88		n = 85		n = 100	
Model 1^c^	0.76 (0.67‐0.86)	<.001	1.00 (0.80‐1.23)	.971	1.10 (0.88‐1.38)	.381	1.19 (0.99‐1.44)	.061
Model 2^d^	0.84 (0.74‐0.96)	.008	1.03 (0.82‐1.28)	.805	1.10 (0.88‐1.38)	.406	1.17 (0.96‐1.41)	.114
MG‐H1								
Cases, n	n = 255		n = 88		n = 85		n = 100	
Model 1^c^	0.70 (0.62‐0.79)	<.001	0.87 (0.70‐1.06)	.171	0.96 (0.77‐1.19)	.680	1.26 (1.06–1.50)	.010
Model 2^d^	0.84 (0.74‐0.97)	.015	0.93 (0.74‐1.16)	.505	1.02 (0.80‐1.29)	.892	1.27 (1.06‐1.54)	.011
Sum of 3 AGEs	n = 255		n = 88		n = 85		n = 100	
Model 1^c^	0.72 (0.63‐0.81)	<.001	0.89 (0.73‐1.10)	.283	1.04 (0.83‐1.29)	.731	1.28 (1.06‐1.54)	.009
Model 2^d^	0.84 (0.73‐0.95)	.008	0.93 (0.75‐1.16)	.548	1.07 (0.85‐1.35)	.546	1.26 (1.04‐1.53)	.016

Abbreviations: CEL, N^ε^‐[1‐carboxyethyl]lysine; CML, N^ε^‐[carboxymethyl]lysine; HCC, hepatobiliary cancer; MG‐H1, N^δ^‐[5‐hydro‐5‐methyl‐4‐imidazolon‐2‐yl]‐ornithine.

^a^ Residuals were computed by a linear regression of the ln‐transformed intake of AGEs on total energy intake and center.

^b^ Dietary intake of AGEs was modeled as a continuous variable per 1 SD increment.

^c^ Model 1: Energy‐adjusted and stratified by sex, age at recruitment in 1‐year categories and center.

^d^ Model 2: Model 1 and additionally adjusted for educational level, body mass index, physical activity (Cambridge index), smoking intensity, lifetime and baseline alcohol intake, coffee intake, self‐reported diabetes and dietary fiber intake.

In fully adjusted models (model 2), these associations were attenuated but remained inversely associated with the risk of HCC, with HRs corresponding to 0.87 (95% CI, 0.76‐0.99), 0.84 (95% CI, 0.74‐0.96) and 0.84 (95% CI, 0.74‐0.97) per 1 SD increment in dietary intake of CML, CEL and MG‐H1, respectively (Table 2).

**FIGURE 1:**
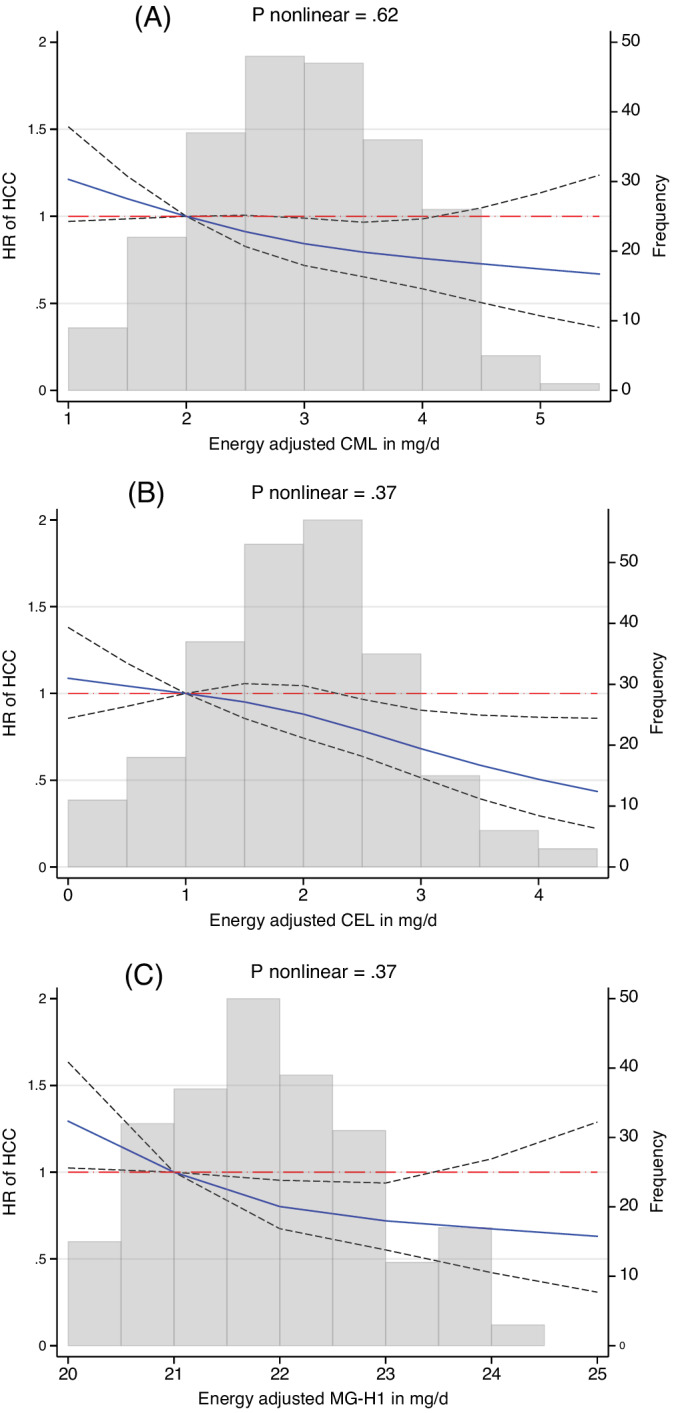
Three knot splines for the association between energy‐adjusted dietary intakes of (A) CML, (B) CEL and (C) MG‐H1 with risk of hepatocellular carcinoma (HCC). CML, N^ε^‐[carboxymethyl]lysine; CEL, N^ε^‐[1‐carboxyethyl]lysine; MG‐H1, N^δ^‐[5‐hydro‐5‐methyl‐4‐imidazolon‐2‐yl]‐ornithine. Hazard ratios (HR) and 95% confidence intervals (black dashed lines) from Cox proportional hazard regression stratified by sex, center and age at recruitment (1‐year categories), and additionally adjusted for educational level, body mass index, physical activity, smoking intensity, lifetime and baseline alcohol intake, coffee intake, self‐reported diabetes and dietary fiber intake [Color figure can be viewed at wileyonlinelibrary.com]

We assessed the shape of the dose‐response association with restricted cubic splines, which confirmed linear inverse relationships between dietary intakes of AGEs and HCC risk (all *P*‐nonlinearity ≥.37) (Figure 1). Associations by tertiles of AGEs intake are shown in Table S2.

There was little evidence for heterogeneity in associations between AGEs and HCC across levels of lifetime alcohol consumption, prevalent diabetes, body weight status, smoking status, geographical region, median age, sex, or plausibility of reporting total energy intake after accounting for multiple testing using Bonferroni correction (all *P*‐interaction ≥0.08) (Figures S4‐S6). We detected no heterogeneity by country for CEL and MG‐H1 (both *I* ^2^ = 0%), and moderate heterogeneity for CML (I^2^ = 54.8%, *P* = .05). Heterogeneity for the latter was likely due to the observed diverging associations (positive but with 95% CI including the null) between CML intake and risk of HCC in Italy and France/Spain as compared to the other countries (Figure S7).

### 3.4 Estimated dietary intake of AGEs and risk of cancers of the bile duct and gallbladder

In minimally adjusted models, CML and MG‐H1 were positively associated with risk of gallbladder cancer with HRs equal to 1.30 (95% CI, 1.07‐1.57) and 1.26 (95% CI, 1.06‐1.50) per 1 SD increment, respectively. These associations remained similar in fully adjusted models (Table 2). In categorical analyses, a linear dose‐response association was evident for MG‐H1 (*P*‐trend = .03) with a HR equal to 1.81 (95% CI, 1.07‐3.06) comparing highest vs lowest tertile of MG‐H1 intake (Table S5). We did not observe heterogeneity by country in associations between any of the three AGEs and gallbladder cancer (all *I* ^2^ ≤ 21%) (Figure S8). There was also no indication of nonlinearity (Figure S9). There was some evidence for an association between higher CEL intake and risk of gallbladder cancer (Table 2).

There was little evidence for associations between higher intakes of CML, CEL, or MG‐H1 and risk of cancers of the intrahepatic or extrahepatic bile duct (Table 2 and Tables S3–S5).

### 3.5 Sensitivity analysis

We also performed a range of sensitivity analyses for the association between dietary AGEs and risk of hepatobiliary cancers, summarized in Table S6. Briefly, the associations of dietary intake of CML, CEL and MG‐H1 with respectively HCC and gallbladder were slightly more pronounced when we excluded cases occurring during the first 2 years of follow‐up. Risk estimates were similar after further adjustment for the Mediterranean dietary pattern and after excluding in turn current smokers and participants reporting heavy alcohol consumption at any point in time. Furthermore, accounting for dietary energy misreporting did not modify associations. In complete case analyses, point estimates of HRs were similar to the primary analysis using a missing value indicator. We used the EPIC nested case‐control study to perform additional adjustment for HBV/HCV and in turn liver function status. The results of these analyses were similar in direction and magnitude to those reported for the entire cohort. Adjustment for main food sources of dietary AGEs (cereals, red and processed meats and cakes and biscuits) instead of the Mediterranean diet did not alter the findings (Table S6), which suggests that other constituents of these foods cannot explain observed associations. We also evaluated whether associations between dietary intakes of AGEs and HCC differed by follow‐up time (Figure S10) by censoring every 2 years. The direction of associations remained, with the strongest inverse effect found censoring at 8 years for MG‐H1.

Similarly, robust results were observed after sensitivity analysis for associations with gallbladder cancer (Table S7).

The sum of the three AGEs (assessed as a continuous variable per 1 SD increment) was inversely associated with the risk of HCC (HR = 0.84, 95% CI, 0.73‐0.95), and positively associated with gallbladder cancer (HR = 1.26, 95% CI, 1.04‐1.53), while no association was observed for intra or extrahepatic bile duct cancers (Table 2).

## 4 DISCUSSION

In this prospective investigation of associations between dietary intake of three well‐characterized AGEs and risk of hepatobiliary cancers, we found that higher intakes of CML, CEL and MG‐H1 were inversely associated with the risk of HCC, and positively associated with the risk of gallbladder cancer. No associations were observed with cancers of the intrahepatic and extrahepatic bile duct. The inverse associations between higher intakes of dietary AGEs and HCC contrast with our hypothesis. Observed associations were, however, robust as further shown after a range of sensitivity analyses. For example, excluding incident cases of HCC ascertained within the first 2 years of follow‐up to account for reverse causation did not alter our findings. There was also little heterogeneity in associations across subgroups of the population as defined by age, body weight status, alcohol consumption, smoking status, prevalent diabetes and geographical region, except for a suggestive heterogeneity by sex. In a subsample of our study population, we were also able to account for hepatitis infection status and liver function with little change in risk associations.

Our findings are consistent with the results of a case‐cohort study among Finnish male smokers, where higher prediagnostic serum concentrations of CML were inversely associated with HCC.^32^ However, there remains an ongoing debate whether dietary AGEs and serum concentrations of AGEs are correlated.^33^, ^34^ Furthermore, a retrospective case‐control study reported higher CML serum levels independent of hepatitis infection status in 90 patients with HCC compared to controls without HCC.^35^ This suggests that hepatitis infection may not confound associations between AGEs exposure and risk of HCC, which confirms our observation whereby adjustment for hepatitis infection did not alter observed associations between AGEs and HCC. We are not aware of published epidemiological studies investigating associations between dietary AGEs intake and risk of cancers of the biliary tract or gallbladder.

An experimental study in a drosophila model found that higher exposure to methylglyoxal (the major precursor of AGEs) increased survival and enhanced resistance to oxidative stress compared to controls.^36^ Considering that a major cause of cancer is damage to deoxyribonucleic acid as a result of oxidative stress,^37^ elevated oxidative stress resistance due to higher AGEs exposure in noncancerous liver cells could offer a potential explanation of our findings with regard to HCC. This is in line with two rodent models where initially induced liver inflammation in high‐AGE fed rodents resolved during the study period and the authors argued that antioxidant mechanisms may have been activated to counter the oxidative stress induced by a high AGE diet.^38^, ^39^ A large proportion of ingested AGEs accumulates in the body.^40^ Importantly, there appear to be large differences in the accumulation of ingested AGEs across tissues and organs.^41^ In a mouse model it has been shown that dietary CML accumulation is particularly high in kidneys and the gut, while it is 20‐30 times lower in the liver.^13^, ^41^ This suggests that dietary AGEs may not accumulate in liver tissue despite being one of the main organs of AGEs metabolism. In contrast, it is conceivable that such adaptations to prolonged AGEs exposure are less developed in the gallbladder. Indeed, in a model of diabetic mice,^42^ AGEs content was significantly higher in the gallbladder of diabetic mice compared to controls, while in the liver opposite effects were observed. These differences in tissue accumulation may therefore partly explain our divergent findings across cancer sites.

The strengths of our study include the investigation of three different AGE compounds and its associations with hepatobiliary cancer subsites, the prospective design, long follow‐up and the large sample size. We performed sensitivity analysis to address potential reverse causation and residual confounding by hepatitis virus infection, liver function, diet, lifestyle and exposure measurement error. Our study also had limitations. First, diet and other lifestyle variables were only available at baseline. However, we conducted extended analyses where we censored results every 2 years to assess the stability of diet consumption over follow‐up, showing relatively stable HCC risk estimates over the follow‐up time (Figure S8). Second, measurement error in collecting dietary intake data and in estimating dietary AGEs exposure, which is also influenced by personal cooking preferences, cannot be excluded. However, we found a positive association between higher intake of CEL, CML and MG‐H1 with weight gain/obesity after an average of 5 years of follow‐up in the same study population, which indicates face validity of our dietary AGE assessment.^24^ The number of cases for cancers of the gallbladder (n = 100), intra (n = 88) and extrahepatic bile duct were limited, which may affect the reliability of risk estimates, and larger studies are warranted to confirm our findings. We were not able to control for other potentially important confounding factors including family history of hepatobiliary cancers, nonalcoholic fatty liver disease, nonalcoholic steatohepatitis, or cirrhosis due to the unavailability of these data.

In conclusion, higher intakes of dietary AGEs were inversely associated with the risk of HCC and positively associated with risk of gallbladder cancer, while no association with intra or extrahepatic bile duct cancer was observed. Our findings with regard to HCC are in contrast to the prevailing hypothesis that dietary AGEs may increase cancer risk. Overall, evidence is still scarce and the reasons for the inverse relationship with the risk of HCC are unknown. Further studies, including those with complementary study designs, are needed to confirm our findings.

## CONFLICT OF INTEREST

None of the authors declared a conflict of interest. Where authors are identified as personnel of the International Agency for Research on Cancer/WHO, the authors alone are responsible for the views expressed in this article and they do not necessarily represent the decisions, policy or views of the International Agency for Research on Cancer/WHO.

## ETHICS STATEMENT

The EPIC cohort was successfully reevaluated by the International Agency for Research on Cancer Ethics Committee in 2017. All participants gave their informed consent. The current study received further approval by the IARC Ethics Committee (IEC Project No. 18‐10).

## Supporting information

**Table S1**. Intake of food groups according to tertiles of dietary intake of advanced glycation endproducts (AGEs).
**Table S2**. Hazard ratios (95% confidence intervals) for hepatocellular cancer according to tertiles of dietary intake of advanced glycation endproducts (AGEs).
**Table S3**. Hazard ratios (95% confidence intervals) for hepatobiliary cancer subsites according to tertiles of dietary CML intake.
**Table S4**. Hazard ratios (95% confidence intervals) for hepatobiliary cancer subsites according to tertiles of dietary CEL intake.
**Table S5**. Hazard ratios (95% confidence intervals) for hepatobiliary cancer subsites according to tertiles of dietary MG‐H1 intake.
**Table S6**. Sensitivity analyses showing hazard ratios (95% confidence intervals) for hepatocellular carcinoma according to dietary intake of advanced glycation endproducts (AGEs).
**Table S7**. Sensitivity analyses showing hazard ratios (95% confidence intervals) for gallbladder cancer according to dietary intake of advanced glycation endproducts (AGEs).
**Figure S1**. Flowchart for participant inclusion criteria.
**Figure S2**. Food group sources of dietary intake of advanced glycation endproducts in the European Prospective Investigation into Cancer and Nutrition (EPIC).
**Figure S3**. Percentage contribution of food groups to CML, CEL and MG‐H1 intake in the European Prospective Investigation into Cancer and Nutrition (EPIC) study.
**Figure S4**. Subgroup analysis showing hazard ratios (HR) and 95% confidence intervals (CI) for hepatocellular carcinoma according to dietary intake of CEL.
**Figure S5**. Subgroup analysis showing hazard ratios (HR) and 95% confidence intervals (CI) for hepatocellular carcinoma according to dietary intake of CML.
**Figure S6**. Subgroup analysis showing hazard ratios (HR) and 95% confidence intervals (CI) for hepatocellular carcinoma according to dietary intake of MG‐H1.
**Figure S7**. Subgroup analysis by country showing hazard ratios (HR) and 95% confidence intervals (CI) for hepatocellular carcinoma according to dietary intake of advanced glycation endproducts (AGEs).
**Figure S8**. Subgroup analysis by country showing hazard ratios (HR) and 95% confidence intervals (CI) for gallbladder cancer according to dietary intake of advanced glycation endproducts (AGEs).
**Figure S9**. Three‐knot spline model for associations between energy‐adjusted dietary intake of AGEs and risk of gallbladder cancer.
**Figure S10**. Associations between dietary AGEs (per 1 SD increment) and HCC censoring every 2 years of follow‐up.

## Data Availability

Data described in the study, code book and analytic code will be made available upon request. For information on how to submit an application for gaining access to EPIC data and/or biospecimens, please follow the instructions at http://epic.iarc.fr/access/index.php
