# Supplementary material for: Dietary intake of advanced glycation endproducts and risk of hepatobiliary cancers: A multinational cohort study
Source: Int J Cancer. 2021 May 6;149(4):854–64. doi: 10.1002/ijc.33612 (PMC8360042; doi:10.1002/ijc.33612)

Supporting Information

Dietary intake of advanced glycation endproducts (AGEs) and risk of hepatobiliary cancers: a multinational cohort study

Ana-Lucia Mayén; Elom K. Aglago; Viktoria Knaze; Reynalda Cordova; Casper G. Schalkwijk; Karl-Heinz Wagner; Krasimira Aleksandrova; Veronika Fedirko; Pekka Keski-Rahkonen; Michael F. Leitzmann; Verena Katzke; Bernard Srour; Matthias B. Schulze; Giovanna Masala; Vittorio Krogh; Salvatore Panico; Rosario Tumino; Bas Bueno-de-Mesquita; Magritt Brustad; Antonio Agudo; María Dolores Chirlaque López; Pilar Amiano; Bodil Ohlsson; Stina Ramne; Dagfinn Aune; Elisabete Weiderpass; Mazda Jenab; Heinz Freisling

Table of Contents

Supplementary Table 1. Intake of food groups according to tertiles of dietary intake of advanced glycation endproducts (AGEs).

Supplementary Table 2. Hazard ratios (95% confidence intervals) for hepatocellular cancer according to tertiles of dietary intake of advanced glycation endproducts (AGEs).

Supplementary Table 3. Hazard ratios (95% confidence intervals) for hepatobiliary cancer subsites according to tertiles of dietary CML intake.

Supplementary Table 4. Hazard ratios (95% confidence intervals) for hepatobiliary cancer subsites according to tertiles of dietary CEL intake.

Supplementary Table 5. Hazard ratios (95% confidence intervals) for hepatobiliary cancer subsites according to tertiles of dietary MG-H1 intake.

Supplementary Table 6. Sensitivity analyses showing hazard ratios (95% confidence intervals) for hepatocellular carcinoma according to dietary intake of advanced glycation endproducts (AGEs).

Supplementary Table 7. Sensitivity analyses showing hazard ratios (95% confidence intervals) for gallbladder cancer according to dietary intake of advanced glycation endproducts (AGEs).

Supplementary Figure 1. Flowchart for participant inclusion criteria.

Supplementary Figure 2. Food group sources of dietary intake of advanced glycation endproducts in the European Prospective Investigation into Cancer and nutrition (EPIC).

Supplementary Figure 3. Percentage contribution of food groups to CML, CEL and MG-H1 intake in the European Prospective Investigation into Cancer and nutrition (EPIC) study.

Supplementary Figure 4. Subgroup analysis showing hazard ratios (HR) and 95% confidence intervals (CI) for hepatocellular carcinoma according to dietary intake of CEL.

Supplementary Figure 5. Subgroup analysis showing hazard ratios (HR) and 95% confidence intervals (CI) for hepatocellular carcinoma according to dietary intake of CML.

Supplementary Figure 6. Subgroup analysis showing hazard ratios (HR) and 95% confidence intervals (CI) for hepatocellular carcinoma according to dietary intake of MG-H1.

Supplementary Figure 7. Subgroup analysis by country showing hazard ratios (HR) and 95% confidence intervals (CI) for hepatocellular carcinoma according to dietary intake of advanced glycation endproducts (AGEs)

Supplementary Figure 8. Subgroup analysis by country showing hazard ratios (HR) and 95% confidence intervals (CI) for gallbladder cancer according to dietary intake of advanced glycation endproducts (AGEs)

Supplementary Figure 9. Three-knot spline model for associations between energy-adjusted dietary intake of AGEs and risk of gallbladder cancer.

Supplementary Figure 10. Associations between dietary AGEs (per 1 SD increment) and HCC censoring every 2 years of follow-up.

Supplementary Table 1. Intake of food groups according to tertiles of dietary intake of advanced glycation endproducts (AGEs)[1] in the European Prospective Investigation into Cancer and nutrition, 1992-2000 (n=450,111).

	CML			CEL			MG-H1		
**Food group (g)**	T1	T2	T3	T1	T2	T3	T1	T2	T3
Red and processed meat	89 ± 44	114 ± 50	151 ± 83	84 ± 38	118 ± 57	167 ± 78	96 ± 47	127 ± 75	146 ± 74
Cereals	148 ± 64	233 ± 93	272 ± 133	184 ± 93	237 ± 123	251 ± 113	166 ± 75	238 ± 113	284 ± 128
Dairy	325 ± 294	369 ± 276	371 ± 221	368 ± 271	347 ± 313	331 ± 197	366 ± 324	350 ± 220	323 ± 195
Fish	26 ± 21	36 ± 26	41 ± 34	27 ± 22	32 ± 23	47 ± 36	27 ± 21	37 ± 27	44 ± 35
Potatoes and other tubers	127 ± 104	111 ± 103	94 ± 69	126 ± 104	103 ± 102	106 ± 66	126 ± 102	111 ± 104	91 ± 64
Vegetables	150 ± 103	160 ± 86	171 ± 124	151 ± 102	162 ± 109	167 ± 107	152 ± 100	157 ± 110	174 ± 111
Legumes	12 ± 25	12 ± 24	12 ± 32	10 ± 22	13 ± 27	13 ± 33	10 ± 22	14 ± 27	14 ± 34
Fruits, nuts and seeds	220 ± 217	226 ± 159	220 ± 245	235 ± 222	213 ± 170	213 ± 242	211 ± 203	210 ± 172	254 ± 257
Egg and egg products	22 ± 20	19 ± 14	17 ± 16	22 ± 19	20 ± 18	17 ± 14	22 ± 19	17 ± 14	18 ± 16
Fat	33 ± 19	31 ± 17	30 ± 16	31 ± 18	33 ± 17	30 ± 18	30 ± 17	32 ± 18	32 ± 18
Sugar and confectionary	70 ± 99	51 ± 68	39 ± 46	64 ± 96	56 ± 76	42 ± 45	68 ± 92	45 ± 49	44 ± 74
Cakes and biscuits	18 ± 21	30 ± 25	62 ± 67	21 ± 20	33 ± 32	64 ± 71	19 ± 22	45 ± 44	58 ± 68
Soups, bouillons	23 ± 44	42 ± 70	48 ± 70	35 ± 58	39 ± 66	41 ± 69	33 ± 57	39 ± 73	44 ± 62
Condiments and sauces	11 ± 13	20 ± 22	23 ± 19	11 ± 15	20 ± 18	26 ± 21	14 ± 18	22 ± 21	21 ± 18

CEL, Nε-[1-carboxyethyl]lysine; CML, Nε-[carboxymethyl]lysine; MG-H1, Nδ-[5-hydro-5-methyl-4-imidazolon-2-yl]-ornithine.

[1] Residuals were computed with a linear regression of the ln-transformed intake of AGEs on total energy intake and center.

All values are mean ± SD.

Supplementary Table 2. Hazard ratios (95% confidence intervals) for hepatocellular cancer according to tertiles of dietary intake of advanced glycation endproducts (AGEs)[1] in the European Prospective Investigation into Cancer and nutrition, 1992-2000 (n=450,111).

	Residuals of AGEs, tertiles			
	T1	T2	T3	P_{trend}
**CML**				
Cases, n	109	70	76	
Model 1 [2]	1 (Reference)	0.68 (0.50 - 0.92)	0.71 (0.53 - 0.96)	0.025
Model 2 [3]	1 (Reference)	0.85 (0.62 - 1.17)	0.88 (0.63 - 1.21)	0.428
**CEL**				
Cases, n	101	87	67	
Model 1 [2]	1 (Reference)	0.79 (0.59 - 1.06)	0.64 (0.47 - 0.87)	0.005
Model 2 [3]	1 (Reference)	0.95 (0.70 - 1.29)	0.75 (0.54 - 1.04)	0.085
**MG-H1**				
Cases, n	121	68	66	
Model 1 [2]	1 (Reference)	0.60 (0.44 - 0.81)	0.54 (0.40 - 0.73)	<0.001
Model 2 [3]	1 (Reference)	0.77 (0.56 - 1.07)	0.73 (0.52 - 1.02)	0.068

CEL, Nε-[1-carboxyethyl]lysine; CML, Nε-[carboxymethyl]lysine; MG-H1, Nδ-[5-hydro-5-methyl-4-imidazolon-2-yl]-ornithine. T, tertiles.

[1] Residuals were computed with a linear regression of the ln-transformed intake of AGEs on total energy intake and center.

[2] Model 1: Energy-adjusted and stratified by sex, center, and age at recruitment in 1-year categories.

[3] Model 2: Model 1 and additionally adjusted for educational level, body mass index, physical activity, smoking intensity, lifetime and baseline alcohol intake, coffee intake, self-reported diabetes, and fiber intake.

Supplementary Table 3. Hazard ratios (95% confidence intervals) for hepatobiliary cancer subsites according to tertiles of dietary CML intake[1], European Prospective Investigation into Cancer and nutrition, 1992-2000 (n=450,111).

	Residuals of AGEs, tertiles			
	T1	T2	T3	P_{trend}
**CML**				
Intrahepatic bile duct	n=36	n=26	n=26	
Model 1	1 (Reference)	0.81 (0.49 - 1.35)	0.78 (0.46 - 1.29)	0.330
Model 2	1 (Reference)	0.86 (0.51 - 1.45)	0.83 (0.49 - 1.42)	0.503
Extrahepatic bile duct	n=28	n=30	n=27	
Model 1	1 (Reference)	1.05 (0.62 - 1.77)	0.96 (0.56 - 1.63)	0.868
Model 2	1 (Reference)	1.09 (0.64 - 1.86)	1.01 (0.57 - 1.77)	0.984
Gallbladder	n=28	n=27	n=45	
Model 1	1 (Reference)	0.96 (0.56 - 1.63)	1.49 (0.92 - 2.40)	0.103
Model 2	1 (Reference)	0.92 (0.54 - 1.59)	1.44 (0.88 - 2.36)	0.146

CML, Nε-[carboxymethyl]lysine. T, tertiles.

[1] Residuals were computed with a linear regression of the ln-transformed intake of AGEs on total energy intake and center.

[2] Model 1: Energy-adjusted and stratified by sex, center, and age at recruitment in 1-year categories.

[3] Model 2: Model 1 and additionally adjusted for educational level, body mass index, physical activity, smoking intensity, lifetime and baseline alcohol intake, coffee intake, self-reported diabetes, and fiber intake.

Supplementary Table 4. Hazard ratios (95% confidence intervals) for hepatobiliary cancer subsites according to tertiles of dietary CEL intake[1], European Prospective Investigation into Cancer and nutrition, 1992-2000 (n=450,111).

	Residuals of AGEs, tertiles			
	T1	T2	T3	P_{trend}
**CEL**				
Intrahepatic bile duct	n=30	n=32	n=26	
Model 1	1 (Reference)	1.04 (0.63 - 1.72)	0.85 (0.50 - 1.44)	0.536
Model 2	1 (Reference)	1.08 (0.65 - 1.81)	0.92 (0.53 - 1.58)	0.757
Extrahepatic bile duct	n=23	n=32	n=30	
Model 1	1 (Reference)	1.27 (0.74 - 2.20)	1.24 (0.71 - 2.15)	0.447
Model 2	1 (Reference)	1.26 (0.72 - 2.20)	1.27 (0.72 - 2.24)	0.414
Gallbladder	n=29	n=27	n=44	
Model 1	1 (Reference)	0.99 (0.58 - 1.68)	1.52 (0.95 - 2.44)	0.084
Model 2	1 (Reference)	0.96 (0.56 - 1.63)	1.43 (0.88 - 2.31)	0.150

CEL, Nε-[1-carboxyethyl]lysine; T, tertiles.

[1] Residuals were computed with a linear regression of the ln-transformed intake of AGEs on total energy intake and center.

[2] Model 1: Energy-adjusted and stratified by sex, center, and age at recruitment in 1-year categories.

[3] Model 2: Model 1 and additionally adjusted for educational level, body mass index, physical activity, smoking intensity, lifetime and baseline alcohol intake, coffee intake, self-reported diabetes, and fiber intake.

Supplementary Table 5. Hazard ratios (95% confidence intervals) for hepatobiliary cancer subsites according to tertiles of dietary MG-H1 intake[1], European Prospective Investigation into Cancer and nutrition, 1992-2000 (n=450,111).

	Residuals of AGEs, tertiles			
	T1	T2	T3	P_{trend}
**MG-H1**				
Intrahepatic bile duct	n=35	n=23	n=30	
Model 1	1 (Reference)	0.70 (0.41 - 1.19)	0.88 (0.54 - 1.44)	0.618
Model 2	1 (Reference)	0.78 (0.45 - 1.36)	1.08 (0.63 - 1.84)	0.785
Extrahepatic bile duct	n=29	n=26	n=30	
Model 1	1 (Reference)	0.86 (0.50 - 1.47)	0.97 (0.58 - 1.62)	0.901
Model 2	1 (Reference)	0.94 (0.53 - 1.64)	1.15 (0.65 - 2.04)	0.638
Gallbladder	n=25	n=29	n=46	
Model 1	1 (Reference)	1.21 (0.70 - 2.07)	1.73 (1.06 - 2.84)	0.029
Model 2	1 (Reference)	1.22 (0.70 - 2.13)	1.81 (1.07 - 3.06)	0.028

MG-H1, Nδ-[5-hydro-5-methyl-4-imidazolon-2-yl]-ornithine. T, tertiles.

[1] Residuals were computed with a linear regression of the ln-transformed intake of AGEs on total energy intake and center.

[2] Model 1: Energy-adjusted and stratified by sex, center, and age at recruitment in 1-year categories.
[3] Model 2: Model 1 and additionally adjusted for educational level, body mass index, physical activity, smoking intensity, lifetime and baseline alcohol intake, coffee intake, self-reported diabetes, and fiber intake.

Supplementary Table 6. Sensitivity analyses showing hazard ratios (95% confidence intervals) for hepatocellular carcinoma according to dietary intake of advanced glycation endproducts (AGEs)[1] in the European Prospective Investigation into Cancer and nutrition, 1992-2000 (n=450,111).

	N (%)	Tertile (T)1	T2	T3	P_{trend}	AGEs intake per 1 SD increments	P_{value}
**CML**							
Model 2	450,111 (100)	1 (Reference)	0.85 (0.62 - 1.17)	0.88 (0.63 - 1.21)	0.428	0.87 (0.76 - 0.99)	0.030
Model S1	442,536 (98)	1 (Reference)	0.85 (0.61 - 1.19)	0.91 (0.65 - 1.28)	0.590	0.95 (0.80 - 1.13)	0.572
Model S2	450,111 (100)	1 (Reference)	0.83 (0.60 - 1.13)	0.82 (0.60 - 1.14)	0.242	0.85 (0.74 - 0.96)	0.012
Model S3	309,258 (69)	1 (Reference)	0.82 (0.55 - 1.25)	0.72 (0.48 - 1.10)	0.129	0.84 (0.71 - 0.99)	0.038
Model S4	407,434 (91)	1 (Reference)	0.83 (0.58 – 1.18)	0.86 (0.61 – 1.22)	0.413	0.90 (0.78 – 1.05)	0.176
Model S5	450,111 (100)	1 (Reference)	0.84 (0.61 - 1.16)	0.86 (0.62 - 1.18)	0.346	0.92 (0.78 - 1.09)	0.335
Model S6	301,987 (67)	1 (Reference)	0.83 (0.57 - 1.23)	0.79 (0.53 - 1.18)	0.252	0.84 (0.71 - 0.99)	0.036
Model S7	407 (79)[2]	1 (Reference)	0.66 (0.36 - 1.21)	0.77 (0.42 - 1.42)	0.400	0.83 (0.63 - 1.10)	0.190
Model S8	324 (53)[2]	1 (Reference)	0.89 (0.44 - 1.81)	0.90 (0.45 - 1.80)	0.764	0.88 (0.64 - 1.22)	0.449
Model S9	407 (79)[2]	1 (Reference)	0.68 (0.39 - 1.20)	0.75 (0.42 - 1.31)	0.310	0.80 (0.62 - 1.04)	0.094
Model S10	450,111 (100)	1 (Reference)	0.87 (0.63 - 1.20)	0.92 (0.66 - 1.28)	0.612	0.88 (0.77 - 1.00)	0.055
Model S11	450,111 (100)	1 (Reference)	0.86 (0.63 - 1.19)	0.90 (0.65 - 1.24)	0.506	0.87 (0.77 - 1.00)	0.046
Model S12	450,111 (100)	1 (Reference)	0.87 (0.63 - 1.19)	0.92 (0.66 - 1.27)	0.600	0.88 (0.77 - 1.01)	0.061

**CEL**							
Model 2	450,111 (100)	1 (Reference)	0.95 (0.70 - 1.29)	0.75 (0.54 - 1.04)	0.085	0.84 (0.74 - 0.96)	0.008
Model S1	442,536 (98)	1 (Reference)	0.96 (0.70 - 1.31)	0.74 (0.52 - 1.04)	0.083	0.86 (0.73 - 1.02)	0.088
Model S2	450,111 (100)	1 (Reference)	0.93 (0.69 - 1.26)	0.72 (0.52 - 1.00)	0.051	0.83 (0.73 - 0.94)	0.005
Model S3	309,258 (69)	1 (Reference)	0.95 (0.64 - 1.41)	0.64 (0.41 - 0.98)	0.042	0.85 (0.72 - 1.00)	0.056
Model S4	407,434 (91)	1 (Reference)	1.01 (0.72 - 1.42)	0.79 (0.55 - 1.14)	0.216	0.85 (0.73 - 0.98)	0.028
Model S5	450,111 (100)	1 (Reference)	0.94 (0.69 - 1.27)	0.73 (0.53 - 1.02)	0.067	0.86 (0.73 - 1.01)	0.070
Model S6	301,987 (67)	1 (Reference)	1.02 (0.71 - 1.46)	0.63 (0.42 - 0.96)	0.032	0.79 (0.67 - 0.93)	0.006
Model S7	407 (79)[2]	1 (Reference)	1.01 (0.56 - 1.81)	0.61 (0.33 - 1.15)	0.128	0.70 (0.53 - 0.92)	0.012
Model S8	324 (53)[2]	1 (Reference)	1.08 (0.55 - 2.12)	0.82 (0.41 - 1.65)	0.575	0.74 (0.54 - 1.01)	0.055
Model S9	407 (79)[2]	1 (Reference)	1.07 (0.62 - 1.84)	0.60 (0.34 - 1.06)	0.078	0.68 (0.53 - 0.88)	0.003
Model S10	450,111 (100)	1 (Reference)	0.97 (0.71 - 1.32)	0.77 (0.55 - 1.07)	0.118	0.85 (0.74 - 0.97)	0.013
Model S11	450,111 (100)	1 (Reference)	0.97 (0.71 - 1.33)	0.78 (0.55 - 1.10)	0.162	0.84 (0.73 - 0.97)	0.019
Model S12	450,111 (100)	1 (Reference)	0.96 (0.71 - 1.30)	0.76 (0.54 - 1.06)	0.102	0.84 (0.74 - 0.96)	0.011
**MG-H1**							
Model 2	450,111 (100)	1 (Reference)	0.77 (0.56 - 1.07)	0.73 (0.52 - 1.02)	0.068	0.84 (0.74 - 0.97)	0.015
Model S1	442,536 (98)	1 (Reference)	0.79 (0.56 - 1.10)	0.73 (0.51 - 1.04)	0.082	0.85 (0.71 - 1.02)	0.075
Model S2	450,111 (100)	1 (Reference)	0.77 (0.56 - 1.06)	0.71 (0.51 - 0.99)	0.046	0.85 (0.74 - 0.96)	

							0.012
Model S3	309,258 (69)	1 (Reference)	0.68 (0.45 - 1.04)	0.65 (0.42 - 1.00)	0.050	0.83 (0.70 - 0.99)	0.033
Model S4	407,434 (91)	1 (Reference)	0.78 (0.54 - 1.11)	0.72 (0.50 - 1.05)	0.084	0.85 (0.74 - 0.99)	0.040
Model S5	450,111 (100)	1 (Reference)	0.76 (0.55 - 1.05)	0.72 (0.51 - 1.00)	0.053	0.84 (0.71 - 1.00)	0.048
Model S6	301,987 (67)	1 (Reference)	0.69 (0.46 - 1.03)	0.69 (0.46 - 1.04)	0.078	0.81 (0.68 - 0.97)	0.021
Model S7	407 (79)[2]	1 (Reference)	0.76 (0.42 - 1.37)	0.54 (0.28 - 1.03)	0.059	0.70 (0.52 - 0.94)	0.016
Model S8	324 (53)[2]	1 (Reference)	0.72 (0.36 - 1.43)	0.55 (0.26 - 1.17)	0.122	0.69 (0.49 - 0.97)	0.034
Model S9	407 (79)[2]	1 (Reference)	0.66 (0.38 - 1.15)	0.60 (0.33 - 1.07)	0.084	0.71 (0.54 - 0.92)	0.010
Model S10	450,111 (100)	1 (Reference)	0.79 (0.57 - 1.09)	0.75 (0.53 - 1.06)	0.098	0.85 (0.74 - 0.98)	0.025
Model S11	450,111 (100)	1 (Reference)	0.77 (0.56 - 1.07)	0.73 (0.52 - 1.03)	0.073	0.85 (0.74 - 0.97)	0.019
Model S12	450,111 (100)	1 (Reference)	0.79 (0.57 - 1.09)	0.76 (0.53 - 1.08)	0.128	0.85 (0.74 - 0.99)	0.037

CML, Nε-[carboxymethyl]lysine; CEL, Nε-[1-carboxyethyl]lysine; MG-H1, Nδ-[5-hydro-5-methyl-4-imidazolon-2-yl]-ornithine; NA, not available.

[1] Residuals were computed by a linear regression of the ln-transformed intake of AGEs on total energy intake and center.

[2] Percentage refers to the proportion of cases in the nested case-control dataset (n=202) compared to the number of cases in full cohort (n=255).

Model 2: main model stratified by sex, center and age in 1-year categories, and adjusted for total energy intake, educational level, body mass index, physical activity, smoking intensity, lifetime and baseline alcohol intake, coffee intake, self-reported diabetes, and fiber intake.

Model S1: model 2 after excluding cancer events that occurred during the first two years of follow-up.

Model S2: model 2 adjusted for the Mediterranean dietary score (mrMDS) instead of fiber intake.

Model S3: model 2 after excluding current smokers.

Model S4: model 2 after excluding subjects reporting heavy drinking at any point in lifetime.

Model S5: model 2 with additional adjustment for under- or over-reporting of total energy intake according to Goldberg.

Model S6: model 2 using a complete case analysis.

Model S7: Odds ratio and 95% confidence interval from conditional logistic regression using the nested case-control dataset with n=204 cases and n=205 matched controls, adjusted for matching factors (incl. sex, age, center), and total energy intake, educational level, body mass index, physical activity, smoking status, alcohol at recruitment, coffee intake, self-reported diabetes, fiber intake, and hepatitis B and C infection status.

Model S8: Odds ratio and 95% confidence interval from conditional logistic regression using the nested case-control dataset excluding those with positive hepatitis B and

C infection status, adjusted for matching factors (incl. sex, age, center), and total energy intake, educational level, body mass index, physical activity, smoking status, alcohol at recruitment, coffee intake, self-reported diabetes, and fiber intake.

Model S9: Odds ratio and 95% confidence interval from conditional logistic regression using the nested case-control dataset with n=204 cases and n=205 matched controls, adjusted for matching factors (incl. sex, age, center), and total energy intake, educational level, body mass index, physical activity, smoking status, alcohol at recruitment, coffee intake, self-reported diabetes, fiber intake, and liver function status: “1” if any was above the clinical threshold (ALT>55 U l−1, AST>34 U l−1, GGT men >64 U l−1, GGT women>36 U l−1, ALP>150 U l−1, albumin<34 g l−1, total bilirubin>20.5 µmol l−1; based on the values provided by the laboratory) vs. “0”.

Model S10: model 2 with additional adjustment for cake & biscuit intake.

Model S11: model 2 with additional adjustment for red meat & processed meat intake.

Model S12: model 2 with additional adjustment for cereal intake.

Supplementary Table 7. Sensitivity analyses showing hazard ratios (95% confidence intervals) for gallbladder cancer according to dietary intake of advanced glycation endproducts (AGEs)[1] in the European Prospective Investigation into Cancer and nutrition, 1992-2000 (n=450,111).

	N (%)	Tertile (T)1	T2	T3	P_{trend}	AGEs intake per 1 SD increments	P_{value}
**CML**							
Model 2	450,111 (100)	1 (Reference)	0.92 (0.54 - 1.59)	1.44 (0.88 - 2.36)	0.146	1.28 (1.05 - 1.56)	0.014
Model S1	442,536 (98)	1 (Reference)	0.95 (0.54 - 1.66)	1.45 (0.87 - 2.42)	0.156	1.23 (0.94 - 1.60)	0.130
Model S2	450,111 (100)	1 (Reference)	0.94 (0.55 - 1.61)	1.46 (0.89 - 2.39)	0.135	1.29 (1.06 - 1.58)	0.011
Model S3	309,258 (69)	1 (Reference)	1.00 (0.52 - 1.89)	1.58 (0.88 - 2.83)	0.127	1.28 (1.02 - 1.62)	0.036
Model S4	407,434 (91)	1 (Reference)	1.04 (0.59 - 1.82)	1.51 (0.90. - 2.52)	0.118	1.28 (1.05 - 1.57)	0.017
Model S5	450,111 (100)	1 (Reference)	0.98 (0.57 - 1.69)	1.56 (0.95 - 2.56)	0.080	1.27 (0.99 - 1.64)	0.063
Model S6	301,987 (67)	1 (Reference)	1.01 (0.50 - 2.04)	1.56 (0.81 - 3.01)	0.185	1.28 (0.98 - 1.68)	0.075
Model S7	450,111 (100)	1 (Reference)	0.91 (0.53 - 1.56)	1.38 (0.83 - 2.29)	0.213	1.27 (1.03 - 1.55)	0.023
Model S8	450,111 (100)	1 (Reference)	0.96 (0.56 - 1.66)	1.51 (0.92 - 2.49)	0.101	1.31 (1.07 - 1.59)	0.008
Model S9	450,111 (100)	1 (Reference)	0.90 (0.52 - 1.55)	1.34 (0.81 - 2.21)	0.261	1.23 (1.00 - 1.53)	0.055
**CEL**							
Model 2	450,111 (100)	1 (Reference)	0.96 (0.56 - 1.63)	1.43 (0.88 - 2.31)	0.150	1.17 (0.96 - 1.41)	0.114

Model S1	442,536 (98)	1 (Reference)	0.96 (0.55 - 1.66)	1.42 (0.86 - 2.35)	0.167	1.21 (0.94 - 1.56)	0.146
Model S2	450,111 (100)	1 (Reference)	0.96 (0.56 - 1.64)	1.44 (0.89 - 2.34)	0.133	1.17 (0.97 - 1.42)	0.099
Model S3	309,258 (69)	1 (Reference)	0.86 (0.46 - 1.59)	1.34 (0.77 - 2.32)	0.298	1.15 (0.92 - 1.44)	0.205
Model S4	407,434 (91)	1 (Reference)	1.01 (0.58 - 1.77)	1.55 (0.94 - 2.56)	0.088	1.20 (0.99 - 1.46)	0.065
Model S5	450,111 (100)	1 (Reference)	1.01 (0.59 - 1.72)	1.53 (0.94 - 2.48)	0.087	1.25 (0.98 - 1.60)	0.074
Model S6	301,987 (67)	1 (Reference)	1.14 (0.59 - 2.19)	1.13 (0.59 - 2.16)	0.707	1.09 (0.84 - 1.42)	0.518
Model S7	450,111 (100)	1 (Reference)	0.94 (0.55 - 1.60)	1.38 (0.85 - 2.25)	0.193	1.15 (0.95 - 1.40)	0.143
Model S8	450,111 (100)	1 (Reference)	1.05 (0.61 - 1.80)	1.67 (1.01 - 2.77)	0.046	1.26 (1.03 - 1.54)	0.024
Model S9	450,111 (100)	1 (Reference)	0.94 (0.55 - 1.61)	1.37 (0.85 - 2.24)	0.200	1.14 (0.94 - 1.38)	0.184
**MG-H1**							
Model 2	450,111 (100)	1 (Reference)	1.22 (0.70 - 2.13)	1.81 (1.07 - 3.06)	0.028	1.27 (1.06 - 1.54)	0.011
Model S1	442,536 (98)	1 (Reference)	1.23 (0.69 - 2.18)	1.78 (1.03 - 3.08)	0.040	1.34 (1.02 - 1.77)	0.034
Model S2	450,111 (100)	1 (Reference)	1.22 (0.71 - 2.12)	1.83 (1.10 - 3.06)	0.020	1.28 (1.07 - 1.54)	0.007
Model S3	309,258 (69)	1 (Reference)	1.00 (0.53 - 1.90)	1.55 (0.85 - 2.83)	0.149	1.25 (1.01 - 1.56)	0.044
Model S4	407,434 (91)	1 (Reference)	1.36 (0.76 - 2.43)	1.91 (1.10 - 3.33)	0.022	1.28 (1.06 - 1.55)	0.012
Model S5	450,111 (100)	1 (Reference)	1.28 (0.74 - 2.23)	1.91 (1.13 - 3.24)	0.016	1.39 (1.07 - 1.81)	0.013

Model S6	301,987 (67)	1 (Reference)	1.20 (0.61 - 2.34)	1.40 (0.71 - 2.77)	0.330	1.19 (0.91 - 1.56)	0.192
Model S7	450,111 (100)	1 (Reference)	1.20 (0.69 - 2.09)	1.75 (1.02 - 2.98)	0.040	1.26 (1.04 - 1.53)	0.016
Model S8	450,111 (100)	1 (Reference)	1.23 (0.71 - 2.14)	1.82 (1.08 - 3.09)	0.025	1.28 (1.06 - 1.55)	0.009
Model S9	450,111 (100)	1 (Reference)	1.18 (0.68 - 2.05)	1.66 (0.96 - 2.87)	0.070	1.23 (1.00 - 1.52)	0.051

CML, Nε-[carboxymethyl]lysine; CEL, Nε-[1-carboxyethyl]lysine; MG-H1, Nδ-[5-hydro-5-methyl-4-imidazolon-2-yl]-ornithine.

[1] Residuals were computed with a linear regression of the ln-transformed intake of AGEs on total energy intake and center.

Model 2: main model stratified by sex, center and age in 1-year categories, and adjusted for total energy intake, educational level, body mass index, physical activity, smoking intensity, lifetime and baseline alcohol intake, coffee intake, self-reported diabetes, and fiber intake.

Model S1: model 2 after excluding cancer events that occurred during the first two years of follow-up.

Model S2: model 2 adjusted for the Mediterranean dietary score (mrMDS) instead of fiber intake.

Model S3: model 2 after excluding current smokers.

Model S4: model 2 after excluding subjects reporting heavy drinking at any point in lifetime.

Model S5: model 2 with additional adjustment for under- or over-reporting of total energy intake according to Goldberg.

Model S6: model 2 using a complete case analysis.

Model S7: model 2 with additional adjustment for cake & biscuit intake.

Model S8: model 2 with additional adjustment for red meat & processed meat intake.

Model S9: model 2 with additional adjustment for cereal intake.

Supplementary Figure 1. Flowchart for participant inclusion criteria.

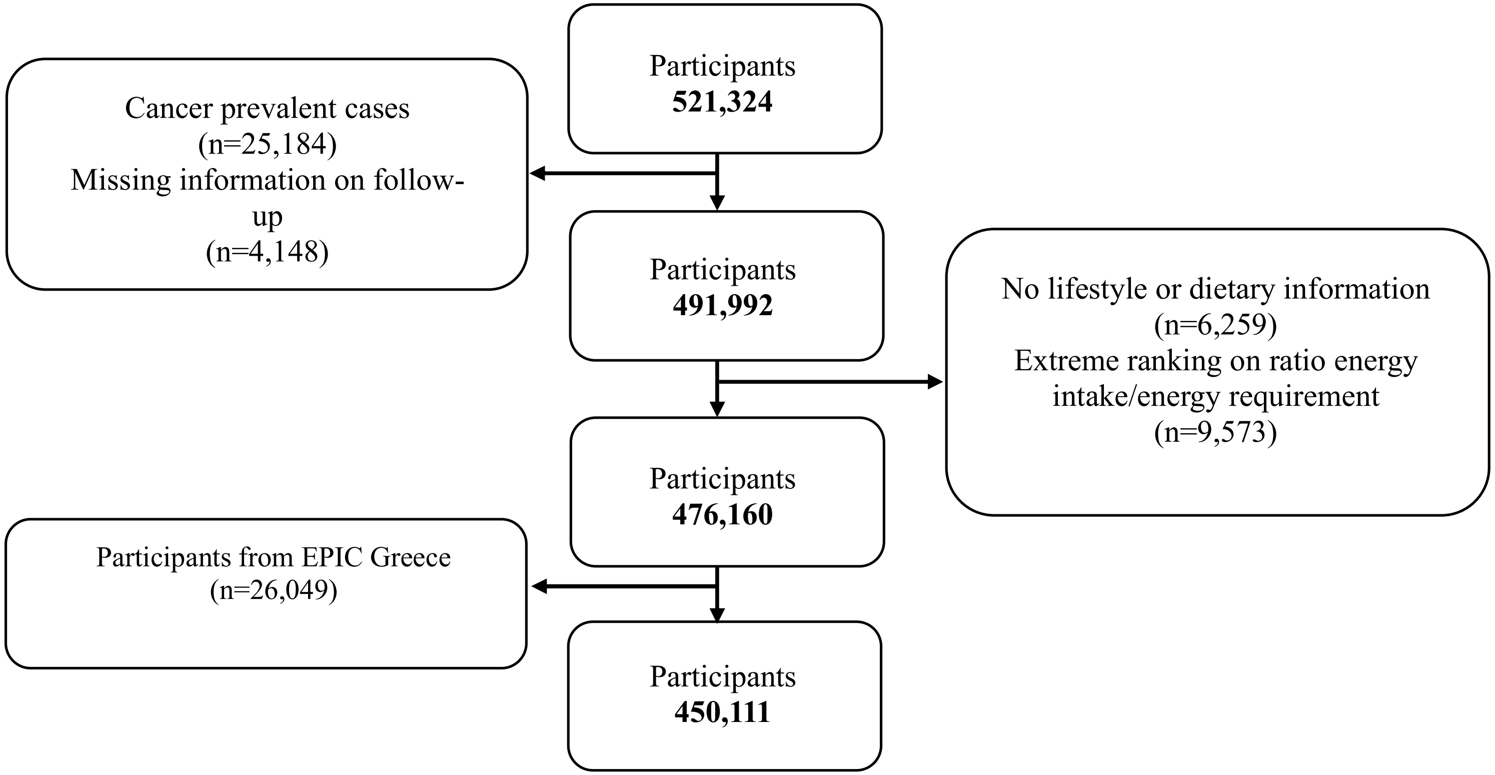

Supplementary Figure 2. Food group sources of dietary intake of advanced glycation endproducts in the European Prospective Investigation into Cancer and nutrition (EPIC).
CML, Nε-[carboxymethyl]lysine; CEL, Nε-[1-carboxyethyl]lysine; MG-H1, Nδ-[5-hydro-5-methyl-4-imidazolon-2-yl]-ornithine.

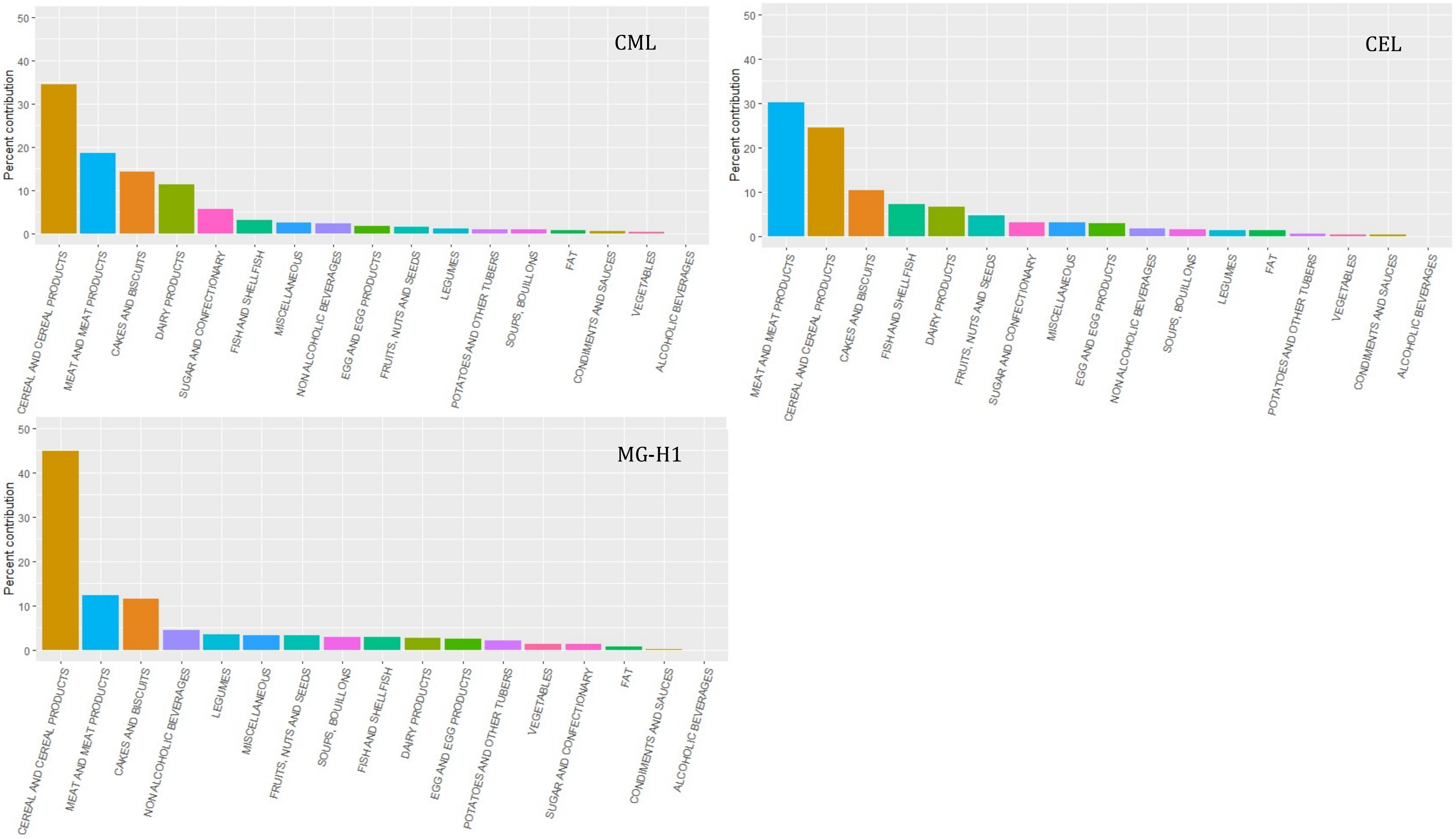

Supplementary Figure 3. Percentage contribution of food groups to CML, CEL and MG-H1 intake in the European Prospective Investigation into Cancer and nutrition (EPIC), by geographical region (North: Sweden, Denmark, and Norway; Central: France, the United Kingdom, the Netherlands, and Germany; South: Italy and Spain).
CML, Nε-[carboxymethyl]lysine; CEL, Nε-[1-carboxyethyl]lysine; MG-H1, Nδ-[5-hydro-5-methyl-4-imidazolon-2-yl]-ornithine.

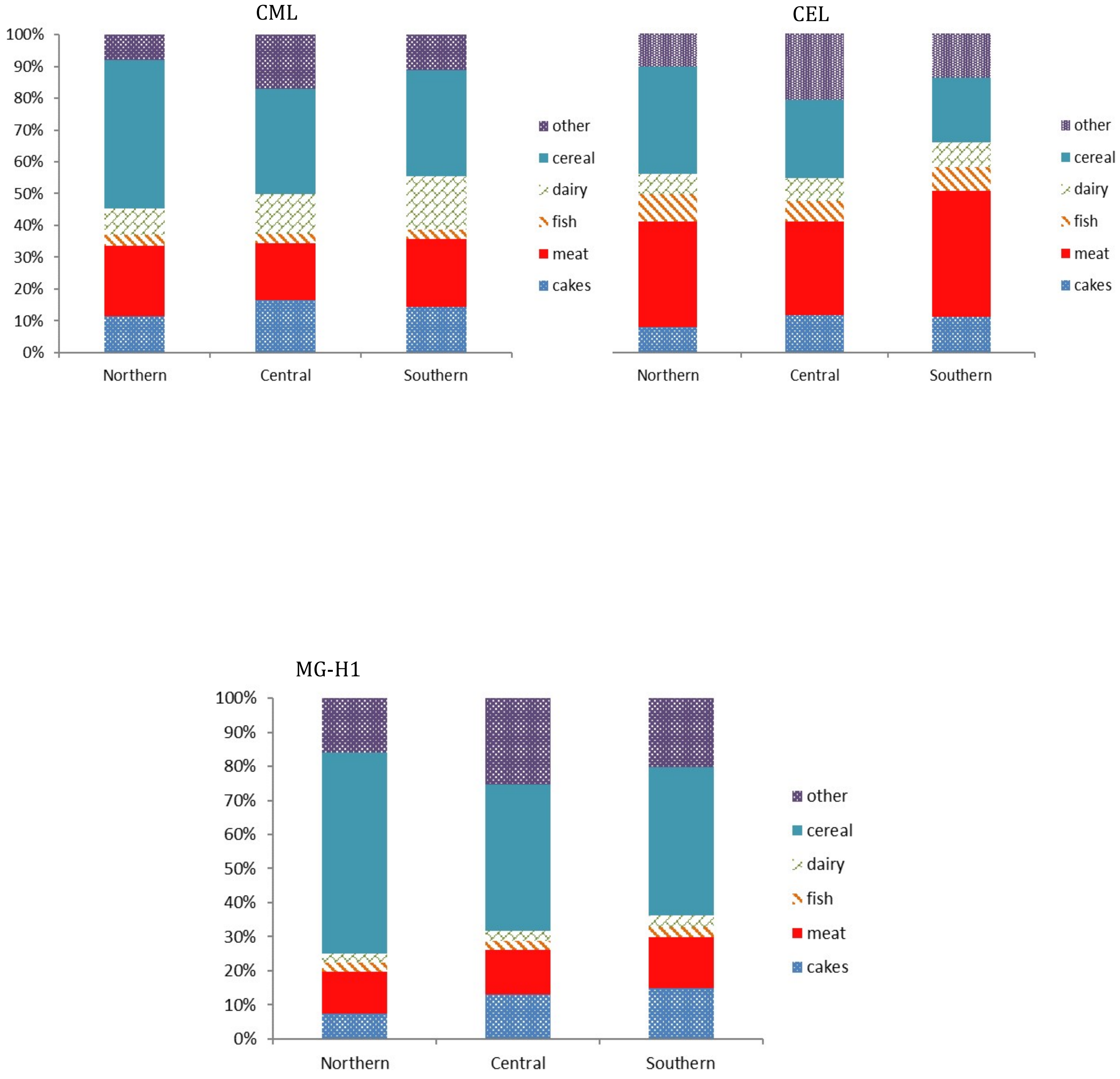

Supplementary Figure 4. Subgroup analysis showing hazard ratios (HR) and 95% confidence intervals (CI) for hepatocellular carcinoma according to dietary intake of advanced glycation endproducts (AGEs)[1] in the European Prospective Investigation into Cancer and nutrition, 1992-2000 (n=450,111).

CML

	Cases	HR(95 %CI)	Pheterogeneity
Sex			
Male	162	0.79 (0.67 - 0.93)	0.170
Female	93	0.98 (0.79 - 1.23)	
Median age			
<median	48	0.97 (0.71 - 1.34)	0.947
>=median	207	0.84 (0.73 - 0.97)	
Lifetime alcohol intake			
Light drinkers	141	0.92 (0.77-1.09)	0.636
Heavy drinkers	55	0.68 (0.50-0.92)	
Goldberg			
Underreporters	45	0.70 (0.51 - 0.96)	0.270
Plausible reporters	185	0.88 (0.75 - 1.04)	
Overreporters	25	0.97 (0.59 - 1.62)	
Diabetes status			
Non-diabetic	195	0.87 (0.75 - 1.01)	0.487
Diabetic	31	0.79 (0.51 - 1.23)	
BMI			
<25	71	0.80 (0.63 - 1.02)	0.890
=25-<30	105	0.86 (0.70 - 1.07)	
>=30	79	1.03 (0.81 - 1.31)	
Smoking			
Never	75	0.90 (0.70 - 1.15)	0.415
Former	79	0.79 (0.62 - 1.00)	
Current	99	0.91 (0.74 - 1.12)	
Geographical region			
Northern	109	0.78 (0.64 - 0.95)	0.687
Central	78	0.79 (0.62 - 0.99)	
Southern	68	1.17 (0.85 - 1.61)	

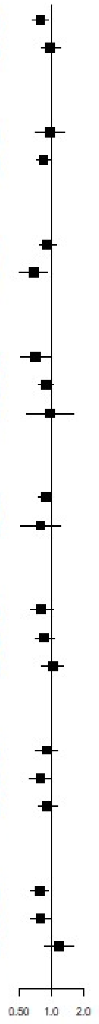

CML, Nε-[carboxymethyl]lysine.
[1] Residuals were computed with a linear regression of the ln-transformed intake of AGEs on total energy intake and center.
Geographical region (North: Sweden, Denmark, and Norway; Central: France, the United Kingdom, the Netherlands, and Germany; South: Italy and Spain).
Model 2: main model stratified by sex, center and age in 1-year categories, and adjusted for total energy intake, educational level, body mass index, physical activity, smoking intensity, lifetime and baseline alcohol intake, coffee intake, self-reported diabetes, and fiber intake.

Supplementary Figure 5. Subgroup analysis showing hazard ratios (HR) and 95% confidence intervals (CI) for hepatocellular carcinoma according to dietary intake of advanced glycation endproducts (AGEs)[1] in the European Prospective Investigation into Cancer and nutrition, 1992-2000 (n=450,111).

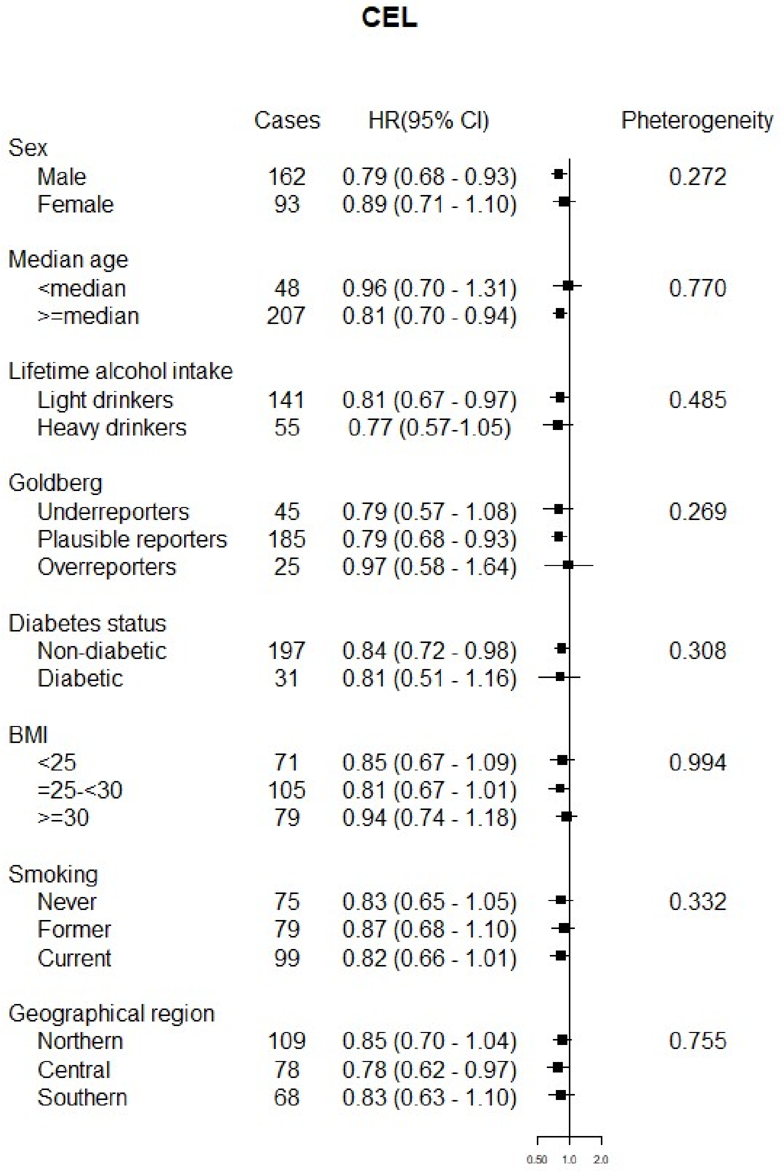

CEL, Nε-[1-carboxyethyl]lysine.

[1] Residuals were computed with a linear regression of the ln-transformed intake of AGEs on total energy intake and center.

Geographical region (North: Sweden, Denmark, and Norway; Central: France, the United Kingdom, the Netherlands, and Germany; South: Italy and Spain).

Model 2: main model stratified by sex, center and age in 1-year categories, and adjusted for total energy intake, educational level, body mass index, physical activity, smoking intensity, lifetime and baseline alcohol intake, coffee intake, self-reported diabetes, and fiber intake.

Supplementary Figure 6. Subgroup analysis showing hazard ratios (HR) and 95% confidence intervals (CI) for hepatocellular carcinoma according to dietary intake of advanced glycation endproducts (AGEs)[1] in the European Prospective Investigation into Cancer and nutrition, 1992-2000 (n=450,111).

MG-H1

	Cases	HR(95% CI)	Pheterogeneity
Sex			
Male	162	0.79 (0.67 - 0.94)	0.237
Female	93	0.92 (0.73 - 1.16)	
Median age			
<median	48	0.84 (0.59 - 1.20)	
>=median	207	0.84 (0.73 - 0.98)	0.723
Lifetime alcohol intake			
Light drinkers	141	0.81 (0.67 - 0.97)	0.991
Heavy drinkers	55	0.81 (0.57 - 1.16)	
Goldberg			
Underreporters	45	0.85 (0.61 - 1.18)	0.746
Plausible reporters	185	0.82 (0.69 - 0.97)	
Overreporters	25	1.01 (0.56 - 1.79)	
Diabetes status			
Non-diabetic	195	0.84 (0.72 - 0.99)	
Diabetic	31	0.86 (0.57 - 1.30)	0.170
BMI			
<25	71	0.74 (0.57 - 0.97)	0.882
=25-<30	105	0.87 (0.70 - 1.09)	
>=30	79	1.02 (0.80 - 1.30)	
Smoking			
Never	75	0.92 (0.72 - 1.19)	0.940
Former	79	0.74 (0.58 - 0.95)	
Current	99	0.90 (0.71 - 1.14)	
Geographical region			
Northern	109	0.81 (0.66 - 0.99)	0.997
Central	78	0.80 (0.62 - 1.03)	
Southern	68	0.92 (0.67 - 1.24)	

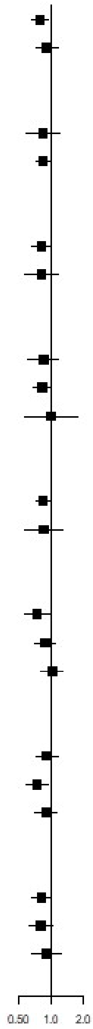

MG-H1, Nδ-[5-hydro-5-methyl-4-imidazolon-2-yl]-ornithine.

[1] Residuals were computed with a linear regression of the ln-transformed intake of AGEs on total energy intake and center.

Geographical region (North: Sweden, Denmark, and Norway; Central: France, the United Kingdom, the Netherlands, and Germany; South: Italy and Spain).

Model 2: main model stratified by sex, center and age in 1-year categories, and adjusted for total energy intake, educational level, body mass index, physical activity, smoking intensity, lifetime and baseline alcohol intake, coffee intake, self-reported diabetes, and fiber intake.

Supplementary Figure 7. Subgroup analysis by country showing hazard ratios (HR) and 95% confidence intervals (CI) for hepatocellular carcinoma according to dietary intake of advanced glycation endproducts (AGEs)[1] in the European Prospective Investigation into Cancer and nutrition, 1992-2000 (n=450,111).

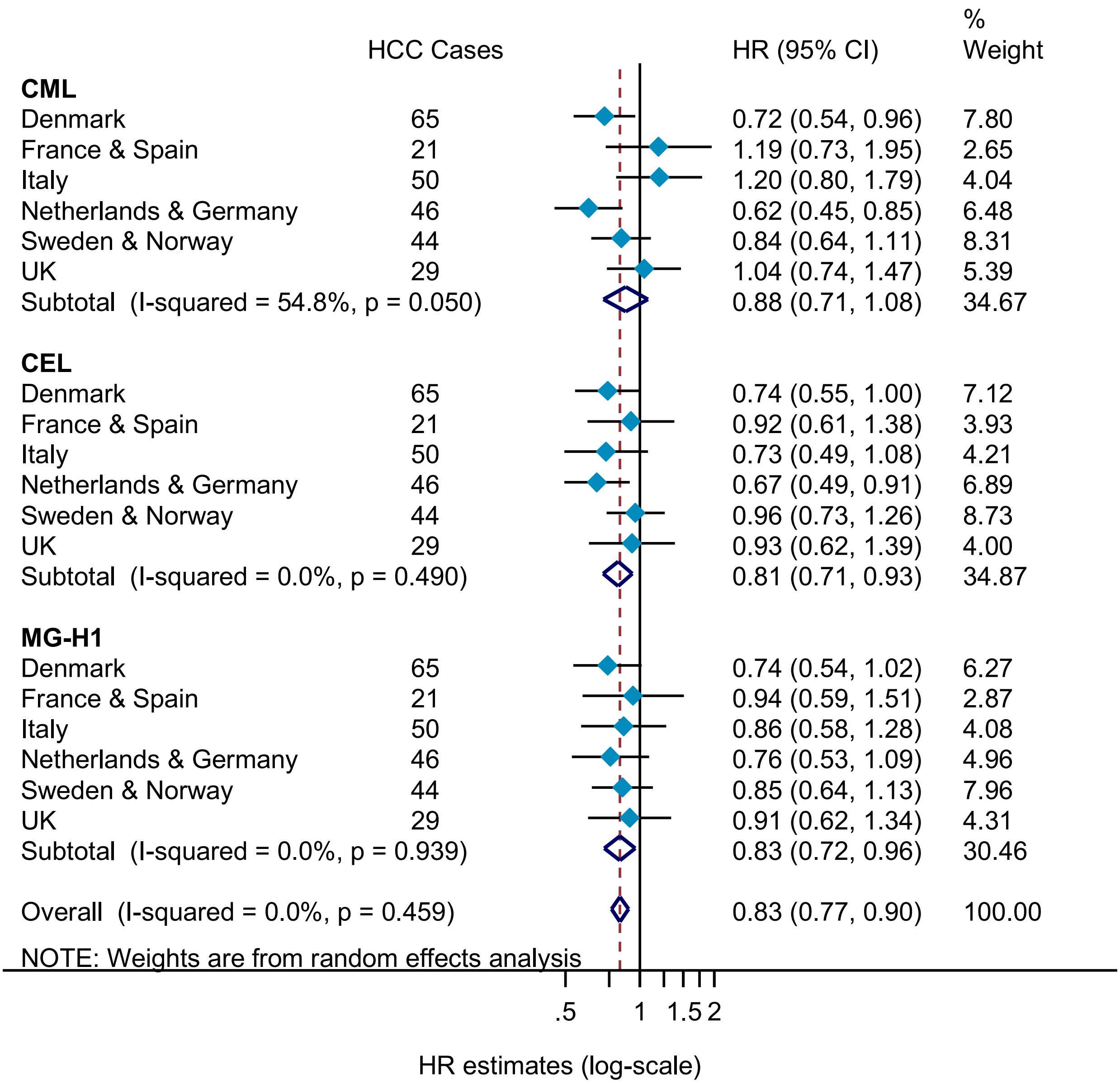

[1] Residuals were computed with a linear regression of the ln-transformed intake of AGEs on total energy intake and center.

Supplementary Figure 8. Subgroup analysis by country showing hazard ratios (HR) and 95% confidence intervals (CI) for gallbladder cancer according to dietary intake of advanced glycation endproducts (AGEs)[1] in the European Prospective Investigation into Cancer and nutrition, 1992-2000 (n=450,111).

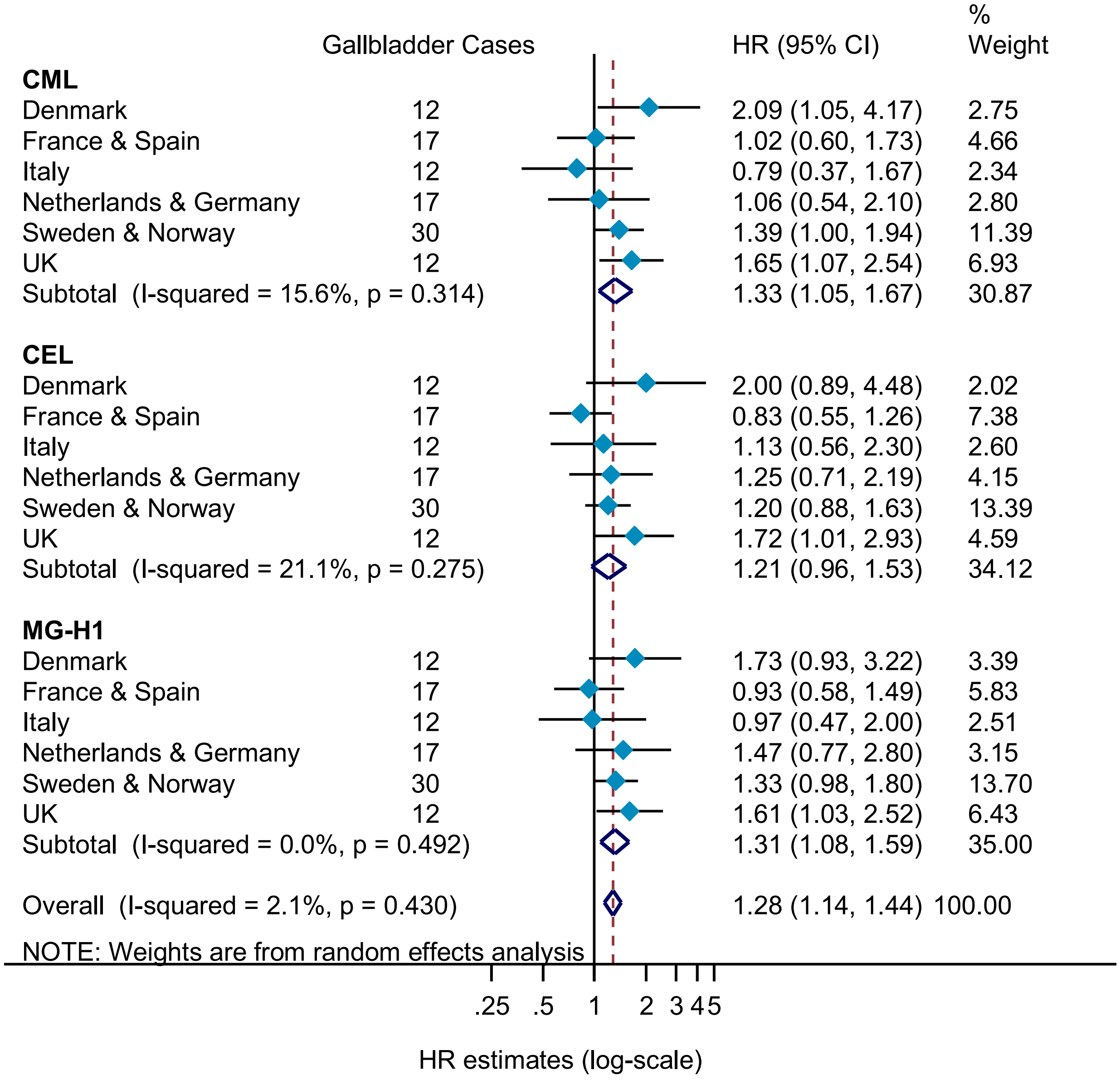

[1] Residuals were computed with a linear regression of the ln-transformed intake of AGEs on total energy intake and center.

Supplementary Figure 9. Three-knot spline model for associations between energy-adjusted dietary intake of AGEs and risk of gallbladder cancer.

CML, Nε-[carboxymethyl]lysine; CEL, Nε-[1-carboxyethyl]lysine; MG-H1, Nδ-[5-hydro-5-methyl-4-imidazolon-2-yl]-ornithine.

Hazard ratio (HR) and 95% confidence intervals (black dotted lines) from Cox proportional hazard regression stratified by sex, center and age at recruitment (1-year categories), and adjusted for educational level, body mass index, physical activity, smoking intensity, lifetime and baseline alcohol intake, coffee intake, self-reported diabetes, total energy intake and dietary fiber intake.

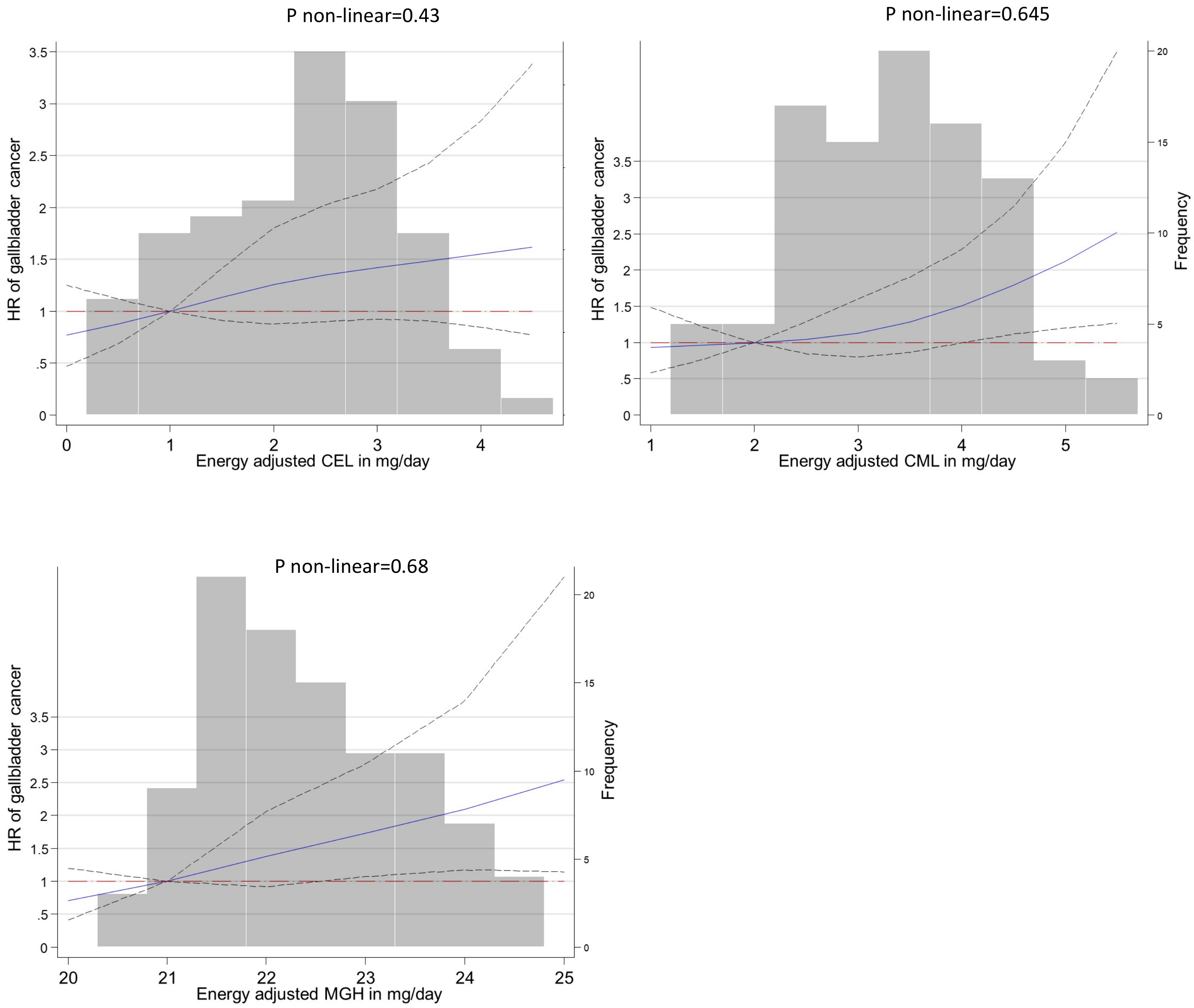

Supplementary Figure 10. Associations between dietary AGEs (per 1 SD increment) and HCC censoring every 2 years of follow-up.
CML, Nε-[carboxymethyl]lysine; CEL, Nε-[1-carboxyethyl]lysine; MG-H1, Nδ-[5-hydro-5-methyl-4-imidazolon-2-yl]-ornithine.
Hazard ratio (HR) and 95% confidence intervals from Cox proportional hazard regression stratified by sex, center and age at recruitment (1-year categories), and adjusted for total energy intake, educational level, body mass index, physical activity, smoking intensity, lifetime and baseline alcohol intake, coffee intake, self-reported diabetes, and dietary fiber intake. HR represent the highest tertile of CML, CEL or MG-H1 consumption.

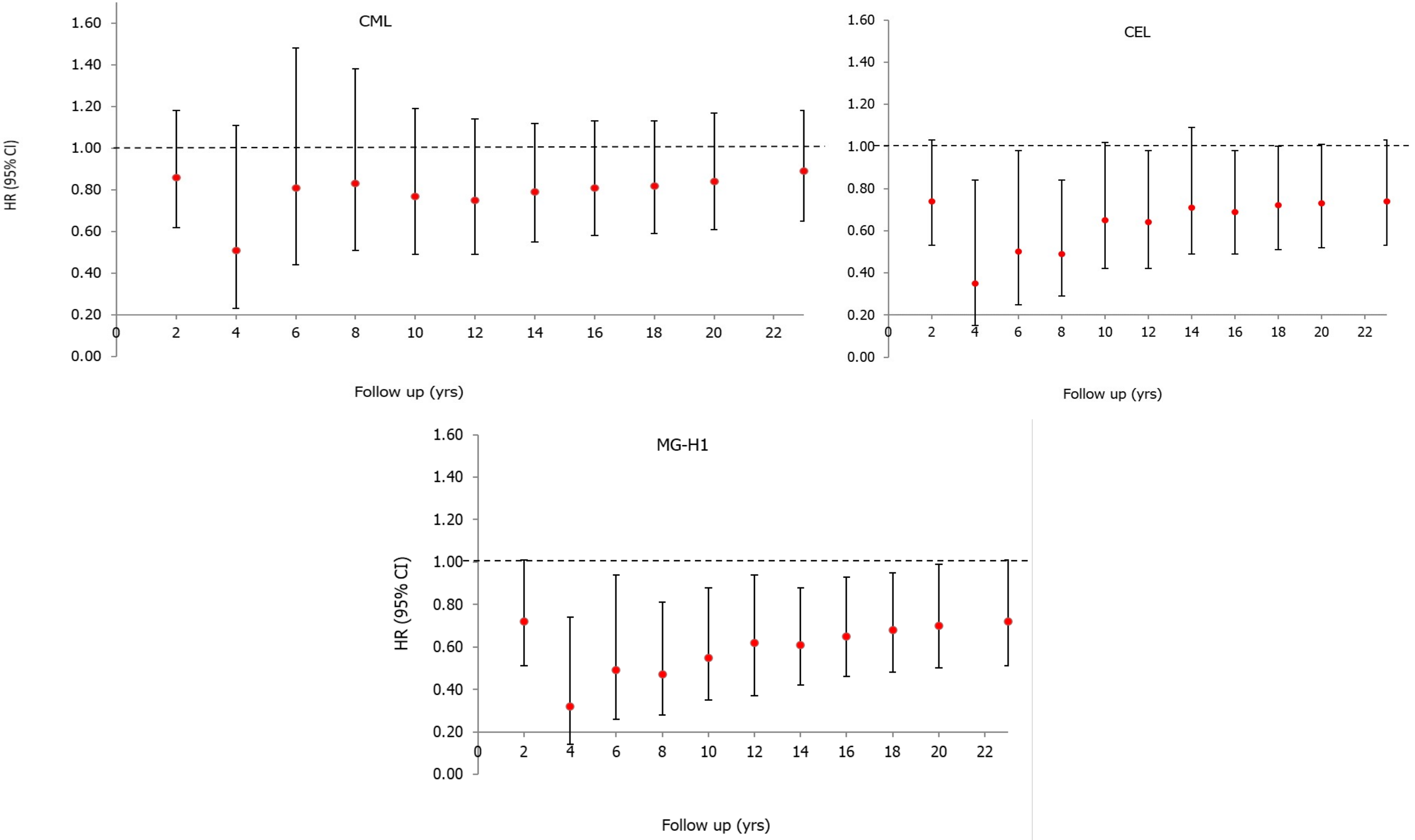

Supplement: Supplementary file 1 — Table S1. Intake of food groups according to tertiles of dietary intake of advanced glycation endproducts (AGEs). Table S2. Hazard ratios (95% confidence intervals) for hepatocellular cancer according to tertiles of dietary intake of advanced glycation endproducts (AGEs). Table S3. Hazard ratios (95% confidence intervals) for hepatobiliary cancer subsites according to tertiles of dietary CML intake. Table S4. Hazard ratios (95% confidence intervals) for hepatobiliary cancer subsites according to tertiles of dietary CEL intake. Table S5. Hazard ratios (95% confidence intervals) for hepatobiliary cancer subsites according to tertiles of dietary MG‐H1 intake. Table S6. Sensitivity analyses showing hazard ratios (95% confidence intervals) for hepatocellular carcinoma according to dietary intake of advanced glycation endproducts (AGEs). Table S7. Sensitivity analyses showing hazard ratios (95% confidence intervals) for gallbladder cancer according to dietary intake of advanced glycation endproducts (AGEs). Figure S1. Flowchart for participant inclusion criteria. Figure S2. Food group sources of dietary intake of advanced glycation endproducts in the European Prospective Investigation into Cancer and Nutrition (EPIC). Figure S3. Percentage contribution of food groups to CML, CEL and MG‐H1 intake in the European Prospective Investigation into Cancer and Nutrition (EPIC) study. Figure S4. Subgroup analysis showing hazard ratios (HR) and 95% confidence intervals (CI) for hepatocellular carcinoma according to dietary intake of CEL. Figure S5. Subgroup analysis showing hazard ratios (HR) and 95% confidence intervals (CI) for hepatocellular carcinoma according to dietary intake of CML. Figure S6. Subgroup analysis showing hazard ratios (HR) and 95% confidence intervals (CI) for hepatocellular carcinoma according to dietary intake of MG‐H1. Figure S7. Subgroup analysis by country showing hazard ratios (HR) and 95% confidence intervals (CI) for hepatocellular carcinoma according [file IJC-149-854-s001.pdf]
